# Supplementary material for: Inter-bacterial mutualism promoted by public goods in a system characterized by deterministic temperature variation
Source: Nat Commun. 2023 Sep 5;14:5394. doi: 10.1038/s41467-023-41224-7 (PMC10480208; doi:10.1038/s41467-023-41224-7)
Supplement: Supplementary file 1 — Supplementary Information [file 41467_2023_41224_MOESM1_ESM.pdf]

Inter-bacterial mutualism promoted by public goods in a system characterized by deterministic temperature variation

Yuxiang Zhao[a], Zishu Liu[a], Baofeng Zhang[b], Jingjie Cai[a], Xiangwu Yao[a], Meng Zhang[a], Ye Deng[c,d], Baolan Hu[a,e, f#]

[a] College of Environmental and Resource Sciences, Zhejiang University, Hangzhou, China

[b] Hangzhou Ecological and Environmental Monitoring Center, Hangzhou, China

[c] CAS Key Laboratory for Environmental Biotechnology, Research Center for Eco-Environmental Sciences, Chinese Academy of Sciences, Beijing, China

[d] College of Resources and Environment, University of Chinese Academy of Sciences, Beijing, China

[e] Zhejiang Province Key Laboratory for Water Pollution Control and Environmental Safety, Hangzhou, China

[f] Key Laboratory of Environment Remediation and Ecological Health, Ministry of Education, College of Environmental Resource Sciences, Zhejiang University, Hangzhou, China

#For correspondence

Baolan Hu

E-mail: blhu@zju.edu.cn

Tel.: 0086-0571-88982340

Fax: 0086-0571-88982340

Supplementary Note 1

Text S1.1 Sampling strategy

The sampling time points were set as follows: Day 00 (initial of composting), Day 05 (maximum mean temperature), Day 12 (halfway point between Day05 and Day20), Day 20 (last day with a mean temperature over 50°C), Day 25 (halfway point between Day20 and Day30), Day 30 (last day of composting). Samples were divided into 6 groups by sampling times for following analyses (i.e., Day00 group, Day05 group, Day12 group, Day20 group, Day25 group, Day30 group).

Text S1.2 Rarefaction analysis

The rarefaction analysis was performed based on the smallest number of sequences measured per sample (i.e. 12,507 reads per sample) (Fig. S1), by using the R packages "*vegan*", "*doBy*" and "*ggalt*". Since the curves of both observed OTU and α-diversity (Shannon index) per sample tended to be flat, the result indicates the sequencing depth of 12,507 reads per sample was sufficient for the amplicon sequencing. We used the mean of three replicates for further analysis of bacterial communities, and if not specifically stated, all 60 samples were included in the analysis. The profiles of composting bacterial communities at the levels of phylum and genus are shown in Fig. S2 and Fig. S3.

Text S1.3 Adonis analysis of bacterial community

We used Adonis analysis to test the differences of bacterial communities between groups, and the grouping method is described in Text S1.1 (i.e., based on 6 sampling time points). 60 samples were included in this analysis. The Adonis analysis was carried out on the basis of Bray–Curtis distance using the R package "*vegan*"[1,2]. Since the number of taxonomically unassigned reads per sample dramatically

increased at the species level, the community composition was recorded at Phylum, Class, Order, Family, Genus, and ASV levels. Results showed that the significant differences could be observed in all tested levels (Table S1).

The significance in Adonis analysis might be caused by homogeneous dispersion or heterogeneous dispersion. Only homogeneous dispersion meets the assumption for Adonis analysis, therefore, we further calculated multivariate homogeneity of group dispersions (variances) to test the homogeneity of dispersion among groups using the R package "*vegan*". No significance ($p > 0.05$) was observed at any of the levels tested, indicating that the result was influenced by the difference in composition between groups (homogeneous dispersion) rather than within groups (heterogeneous dispersion) (Table S2). Thus, the significant differences obtained by Adonis analysis met its assumption, and were reliable. Thus, bacterial communities were significantly different at all tested levels when grouped by sampling time points.

Text S1.4 Pairwise Adonis analysis of Shannon index and Chao1 index

To explore whether there were significant differences between the Shannon and Chao1 indices at different sampling time points, we tested the pairwise Adonis between different groups using the R packages "*vegan*" and "*pairwiseAdonis*". The results showed no significant difference between all paired groups (Table S3), although the means showed a trend of decrease and then recovery (Fig. S4). Further correlation analysis showed a weak negative correlation between positive cohesion and diversity ($p > 0.05$).

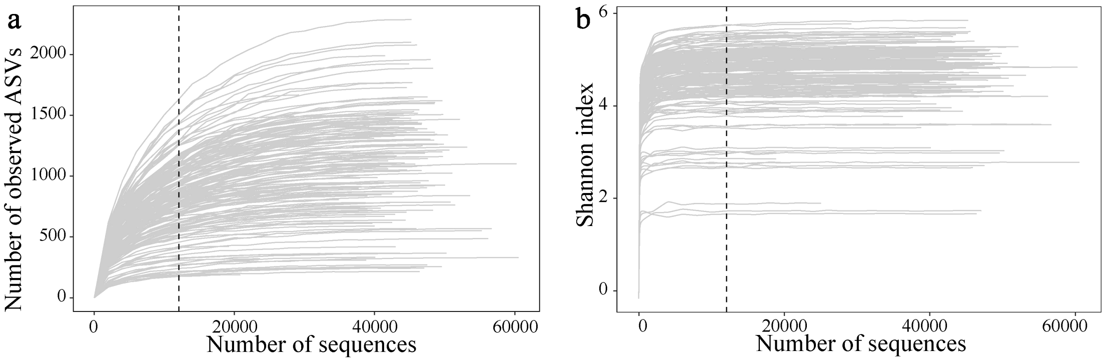

Fig. S1. Rarefaction curves. a) The observed ASVs per sample. b) The α-diversities per sample (Shannon index), which were calculated based on ASV data. Vertical dash line indicates 12057 reads which was smallest number of sequences per sample.

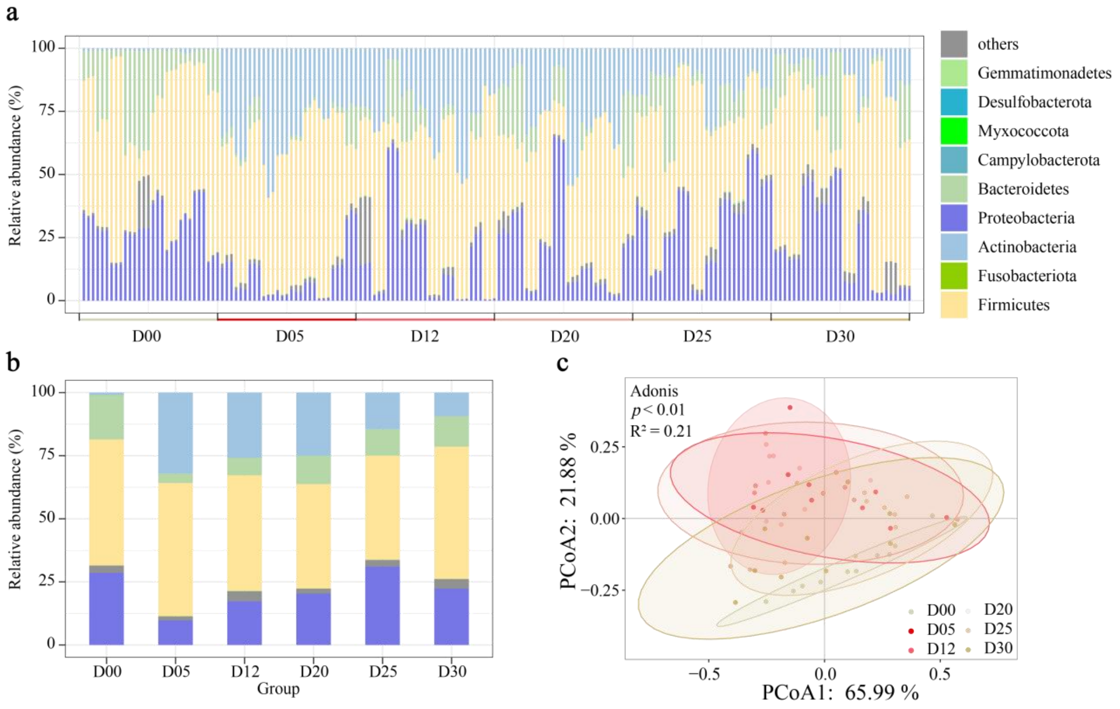

Fig. S2. The overall community structure of the composting bacteria with 10 most abundant taxa at the phylum level. **a)** Overview of 10 most abundant phyla in the 180 samples. **b)** The abundance of 10 most abundant phyla in the 6 groups during the sampling period. **c)** The PCoA analysis of the 6 groups at the phylum level.

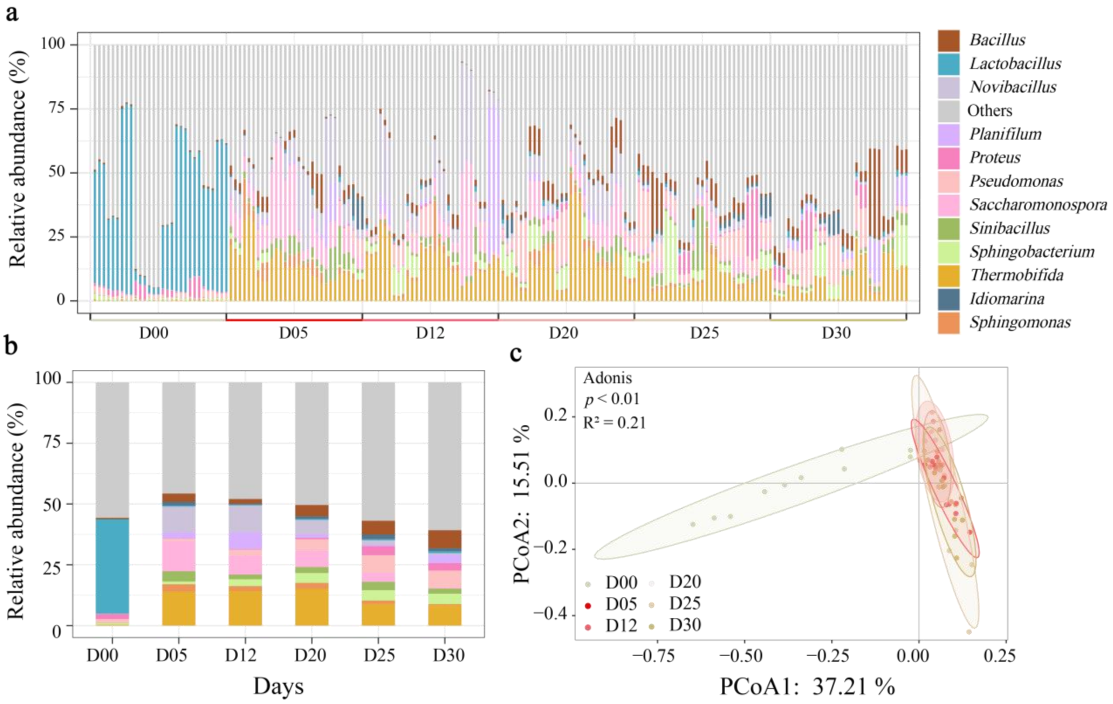

Fig. S3. The overall community structure of the composting bacterial with 12 most abundant taxa at the genus level. **a)** The overview of 12 most abundant genera in 180 samples. **b)** The abundance of 12 most abundant genera in 6 groups set during the sampling period. **c)** The PCoA analysis with 6 groups at the genus level.

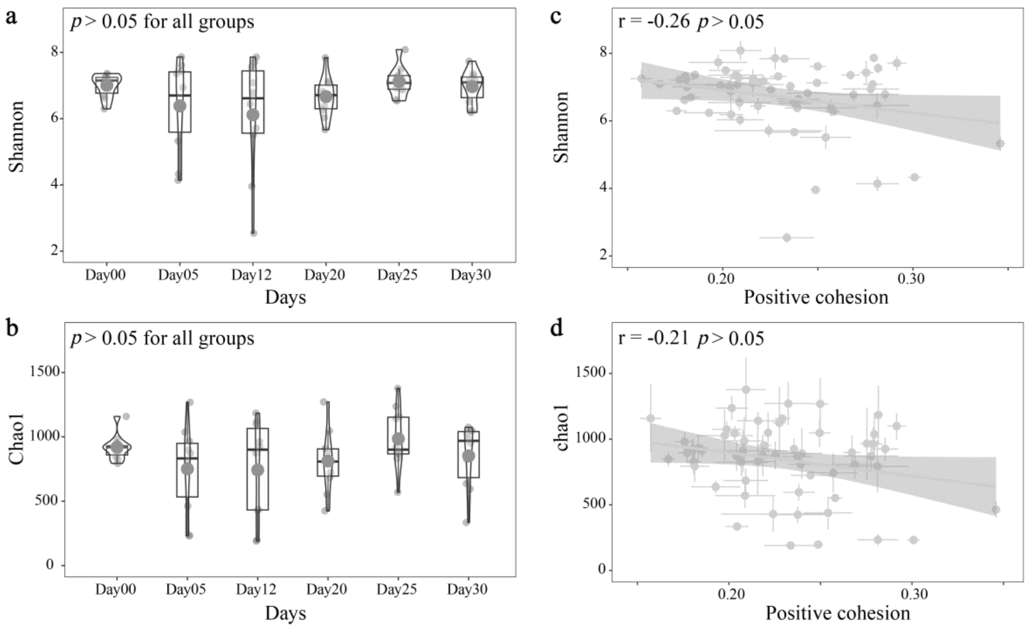

Fig. S4. Variation in α-diversity between sampling times. a) Shannon index. In the boxplots of panels, hinges indicate the 25th, 50th, and 75th percentiles, whiskers indicate 1.5 × interquartile ranges, and dots indicate values of individual samples (n = 10 biologically independent samples for each day). b) Chao1 index (n = 10 biologically independent samples for each day). c) Relationship between Shannon index and positive cohesion (n = 60 biologically independent samples). Data are presented as mean values +/- SEM. d) Relationship between Chao1 index and positive cohesion (n = 60 biologically independent samples). Data are presented as mean values +/- SEM. Grey shading denotes the 95% confidence intervals.

Table S1 Adonis analysis of bacterial community

Group pairs	Sum.sq	R2	F	Pr(>F)	Significant
Phylum	1.2589	0.2093	2.8595	0.01	Yes
Class	1.3042	0.2130	2.9231	0.01	Yes
Order	3.1258	0.4276	8.0693	0.001	Yes
Family	2.3773	0.3849	6.7573	0.001	Yes
Genus	1.8565	0.3623	6.1355	0.001	Yes
ASV	63841823.3	0.2194	3.0361	0.001	Yes

Table S2 Multivariate homogeneity of groups dispersions (variances) of bacterial community

Group pairs	Sum.sq	Mean.sq	F	Pr(>F)	Significant
Phylum	0.1098	0.0220	1.2314	0.2750	No
Class	0.1051	0.0210	1.2319	0.3120	No
Order	0.1082	0.0216	1.3991	0.2380	No
Family	0.0944	0.0189	1.2074	0.3290	No
Genus	0.0801	0.0160	1.1798	0.3340	No
ASV	5140395	1028079	1.2093	0.3320	No

Table S3 Pairwise Adonis analysis of Shannon index and Chao1 index

Diversity	Group pairs	Sum.sq	R2	F	Pr(>F)	Significant
Shannon index	Day00 vs Day05	0.0162	0.1155	2.3499	0.1630	No
	Day00 vs Day12	0.0372	0.1252	2.5755	0.1120	No
	Day00 vs Day20	0.0035	0.1223	2.5093	0.1390	No
	Day00 vs Day25	0.0003	0.0193	0.3549	0.5520	No
	Day00 vs Day30	0.0001	0.0035	0.0640	0.8130	No
	Day05 vs Day12	0.0052	0.0137	0.2497	0.7000	No
	Day05 vs Day20	0.0051	0.0359	0.6705	0.4140	No
	Day05 vs Day25	0.0204	0.1390	2.9061	0.1250	No
	Day05 vs Day30	0.0142	0.0987	1.9708	0.1840	No
	Day12 vs Day20	0.0199	0.0680	1.3126	0.3250	No
	Day12 vs Day25	0.0431	0.1407	2.9468	0.0860	No
	Day12 vs Day30	0.0344	0.1143	2.3232	0.1440	No
	Day20 vs Day25	0.0058	0.1719	3.7375	0.0680	No
	Day20 vs Day30	0.0026	0.078	1.5225	0.2020	No
	Day25 vs Day30	0.0006	0.0289	0.5354	0.4810	No
Chao1 index	Day00 vs Day05	0.1096	0.1425	2.9915	0.1170	No
	Day00 vs Day12	0.1529	0.1529	3.2484	0.0720	No
	Day00 vs Day20	0.0313	0.1211	2.4802	0.1310	No
	Day00 vs Day25	0.0040	0.0241	0.4439	0.5540	No
	Day00 vs Day30	0.0184	0.0577	1.1018	0.3300	No
	Day05 vs Day12	0.0057	0.0039	0.0698	0.8650	No
	Day05 vs Day20	0.0338	0.0387	0.7251	0.4370	No
	Day05 vs Day25	0.1214	0.1357	2.8265	0.0840	No
	Day05 vs Day30	0.0396	0.0416	0.7806	0.3570	No
	Day12 vs Day20	0.0656	0.0600	1.1498	0.3200	No
	Day12 vs Day25	0.1609	0.1434	3.0144	0.0870	No
	Day12 vs Day30	0.0696	0.0595	1.1395	0.2740	No
	Day20 vs Day25	0.0462	0.1195	2.4422	0.1120	No
	Day20 vs Day30	0.0035	0.0073	0.1318	0.8050	No
	Day25 vs Day30	0.0268	0.0609	1.1674	0.2920	No

Supplementary Note 2

Text S2.1 Bacterial community composition

Bacterial communities are influenced by both deterministic and stochastic processes. Deterministic processes include environmental selection and various biological interactions. Therefore, before exploring the effects of microbial interactions and environmental filtering on the composting community, it is necessary to reveal the bacterial community composition and determine whether the composting community is affected by deterministic factors[3]. To quantify various ecological processes (particularly mean community assembly processes), The phylogenetic-bin-based analysis (iCAMP) and QPEN analyses were calculated, respectively[3]. In detail, the obtained ASVs were firstly classified into various bins based on phylogenetic relationships. The author-recommended phylogenetic distance cutoff (d_s = 0.2) was used. The bin size limitation (n_{min}) was checked and set to 24. The beta Net Relatedness Index (βNRI) were calculated to determine the main process for each bin. For each bin, the pairwise comparisons with $|\beta NRI| > 1.96$ were regarded as a percentage of deterministic process, with $\beta NRI < -1.96$ governed by homogeneous selection and $\beta NRI > 1.96$ by heterogeneous selection. Afterward, the taxonomic diversity (RC_{Bray}) was further calculated to separate the stochastic process ($|\beta NRI| \leq 1.96$). Fractions with $RC_{bray} > 0.95$ was considered homogenizing dispersal, while those with $RC_{bray} < -0.95$ were considered dispersal limitation. The remaining portion ($|\beta NRI| \leq 1.96$, $|RC_{bray}| \leq 0.95$) were designated as "drift". Finally, the proportions of the various processes in all bins were abundance weighted and summarized. Meanwhile, the beta mean nearest taxon distance (βMNTD) and beta nearest taxon index (βNTI) were further calculated (QPEN analysis), which are deemed to be more valid for the community-wide-based analysis[4]. All analyses were

performed with the iCAMP package in R (v.4.2.0)[4,5]. Based on iCAMP analysis, dispersal limitation (DL) and homogeneous selection (HoS) were more important than other processes in bacterial community assembly with average relative importance of 34.9% and 64.3% (Fig. S6a), respectively. Thus, according to the iCAMP result, the composting process was regulated by deterministic factors. The results obtained by QPEN were consistent with iCAMP (Fig. S6b) in that composting process was regulated by deterministic factors. The numerical difference between iCAMP and QPEN might be caused by the fact that iCAMP was the mean after bins and smaller taxa are susceptible to stochastic processes. Overall, these results showed that the composting bacterial community is determined by deterministic processes. Thus, it is valuable to further analyse the effects of biotic and abiotic factors on composting bacterial communities.

Text S2.2 Taxon/column shuffling null model

We used the "taxon/column shuffling" null model, as recommended, to calculate the positive and negative cohesion[6]. During each iteration, one taxon was designated as the "focal taxon". For each taxon besides the focal taxon, abundances in the null matrix were permuted from their abundance distribution across all the samples. Then, we calculated Pearson correlations between the focal taxon and the randomized other taxa. We iterated through this process of calculating pairwise correlations between the focal taxon and all other taxa 999 times. The median correlations from these 999 randomizations were called the "expected" correlations for the focal taxon. We recorded the median value as the "expected" correlation, rather than the mean value, because distributions were skewed toward larger values. Thus, a greater proportion of the distribution fell within one standard deviation of the median, as compared with within one standard deviation of the mean. We repeated this process for each taxon as

the focal taxon, which resulted in a matrix of expected taxon correlations. Finally, we subtracted the expected taxon correlations from their paired observed taxon correlations, thereby producing a matrix where each value was an observed minus expected correlation for the given pair of taxa.

Text S2.3 Importance of 'potential positive interaction' in shaping the composting bacterial community.

Theoretically, deterministic community assembly could be driven mainly by two ecological processes: environmental selecting and biotic interaction[7,8]. Unraveling the importance of microbial interactions in contributing to ecological community assembly is a great challenge in ecology, particularly in microbial ecology[5]. Although various theoretical approaches have been developed over the past century, to our knowledge, there is no method yet available to precisely describe the contributions of "microbial interaction" in complex or natural microbial communities[9]. Species co-occurrence- and correlation- based on statistical methods are the most widely used methods for discerning microbial interactions in community ecology. Even though a number of challenges regarding the use of co-occurrence analysis for microbial ecology studies have been raised, with cautious usage and meaningful interpretations, it can still contribute greatly to ecological research. We used various co-occurrence approaches (i.e. cohesion analysis and network analysis) and different statistical approaches to determine the effect of environmental filtering and microbial interaction on shaping the composting microbial community[9].

Firstly, we determined how environmental selection influenced the bacterial community. Decision Curve Analysis (DCA) was carried out to decide whether Canonical correlation analysis (CCA) or Redundancy analysis (RDA) should be used. The first axis of DCA analysis was over 4 (DCA1= 4.4), therefore CCA analysis was

chosen (Fig. S7a). CCA analysis showed that the tested composting environmental factors could explain 45.4% of the responses by the composting community to the increasing of temperature (Fig. S7b). Further variation partitioning analysis (VPA) indicated that more than 64.4% could be explained by the changes of environmental factors and potential microbial interactions (characterised by cohesion and network) (Fig S7c). Sampled piles and sampled environment conditions had less effect on the microbial community (6.4%). Thus, these results implied the importance of microbial interactions in shaping the composting microbial community.

As both environment filtering and microbial interaction could cause the correlations between taxa, we constructed 6 networks using 10 samples from the same sampling time points (i,e., each network is a representative of that sample point) (Fig. S8). Based on these networks, we further used Goberna's method to confirm the influence of various processes to the links between taxa (i.e. microbial interactions, environmental selecting, dispersal limitation and other processes) (Fig. S9). Our results indicated that microbial interactions were the primary driver (on average 71.4 % of the links), rather than dispersal limitation (3.8 %) or environmental filtering (25.0 %). Moreover, we also used the link test for environmental filtering (LTEF) to confirm the relationship between environmental filtering and the observed network links (Fig. S10). We used $|r| > 0.8$ as cutoff but no significant links could be observed. Thus, we lowered the $|r|$ to 0.6 and detected only < 5 % taxon-taxon-environment pairs among all the links. This showed that the links occurring in the network might be caused by real bacterial interactions. Overall, bacterial interactions were one of the main drivers to drive the microbial community.

We further tested the correlations between positive (negative) interaction (characterized by cohesion index) and community assembly and functional potential

to reveal their importance for the composting bacterial community. Community assembly was characterized by the proportions of Hos and DL. Functional potential was characterized by the abundance for 54 functional genes (including carbon degradation, carbon fixation and nitrogen cycling) (see Methods for details) (Fig. S11). Results indicated that positive cohesion was highly associated with both the HoS and DL processes rather than negative cohesion, which showed the importance of positive cohesion to bacterial assembly (Fig. S12). Meanwhile, positive cohesion was also highly associated with the abundance of functional genes (Fig. S11). However, no significant differences could be observed between negative cohesion and the abundance of functional genes (Fig. S13). Together, these results indicated that potential positive interactions (characterized by positive cohesion) was one of the main drivers of the bacterial community. Therefore, focusing on positive cohesion was necessary.

We measured various major environmental factors during the entire composting process that were found to be potential drivers of composting microbial communities in a previous study, including temperature, pH, moisture content (MC), electrical conductivity (EC), E465 / E665 (i.e, the structure of humic acid), ammonia concentration, nitrate concentration, humus content, germination index (GI value, reflecting the phytotoxicity of compost), cellulose content, hemicellulose content, ligin content, starch content (i.e. the four main types of organic matter in food waste composting), total nitrogen, total organic carbon, total organic carbon / total nitrogen ratio (Supplementary Data 11). Indeed, as we did not test all the physicochemical indices of the composting process we cannot rule out the importance of other unmeasured environmental variables (e.g., micronutrients, bioactive molecules, and many specific edaphic factors). Thus, to avoid over-interpretation and confusion, we

could only describe the calculated positive correlations as “potential positive interaction”.

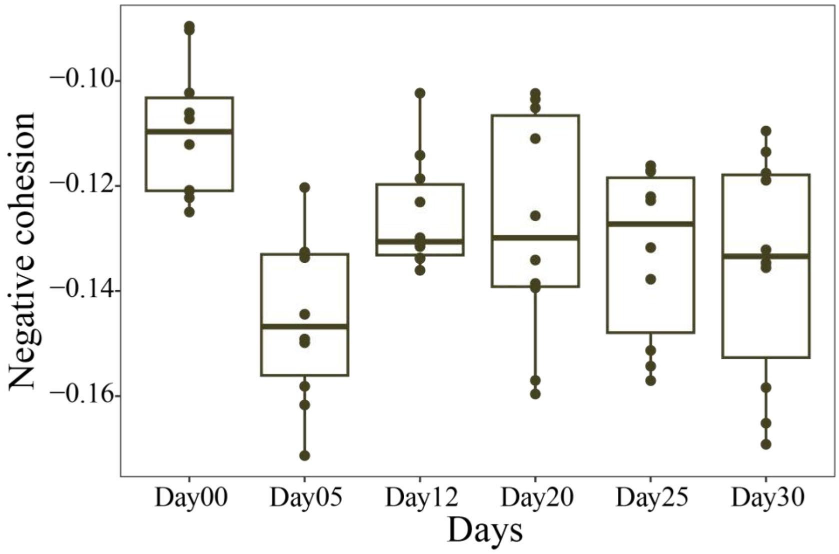

Fig. S5. Calculation of negative cohesion. In the boxplots of panels, hinges indicate the 25th, 50th, and 75th percentiles, whiskers indicate 1.5 × interquartile ranges, and dots indicate values of individual samples (n = 10 biologically independent samples for each day).

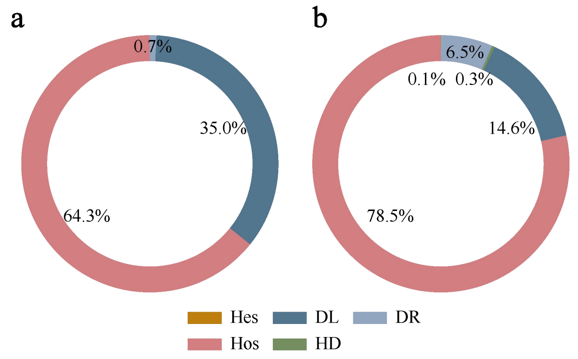

Fig. S6. The overview of composting bacterial assembly. a) Results calculated by iCAMP analysis. b) Results calculated by QPEN analysis. The bacterial community assembly process includes deterministic processes (homogeneous selection (HoS), heterogeneous selection (HeS)) and stochastic processes (homogenizing dispersal (HD), dispersal limitation (DL), and drift (DR)).

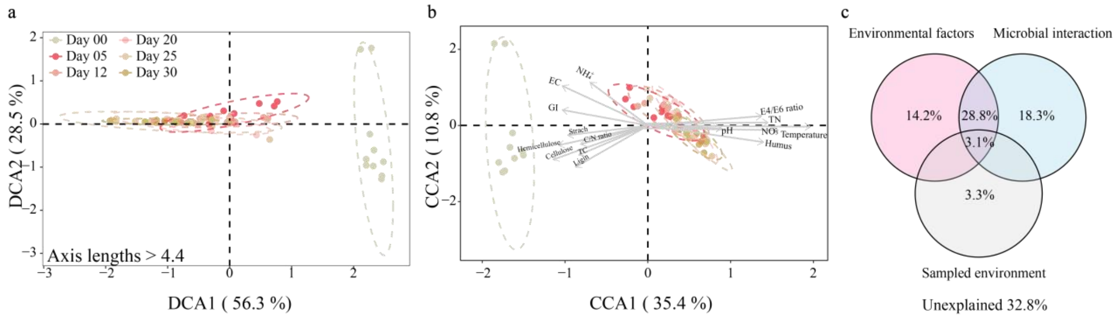

Fig. S7. The importance of environmental factors to the succession of the compost microbiome. **a)** Decision Curve Analysis (DCA). The compost microbiome is characterized by ASVs levels and grouped by sampling time points (n = 10 biologically independent samples for each day). **b)** CCA analysis between the compost microbiome and environmental factors. The compost microbiome is characterized by ASVs levels. The environmental factors included temperature, pH, moisture content (MC), electrical conductivity (EC), E465 / E665 (indicating the structure of humic acid), ammonia concentration, nitrate concentration, humus content, germination index (GI), cellulose content, hemicellulose content, lignin content, starch content, total nitrogen, total organic carbon, total organic carbon / total nitrogen ratio. The model was significant ($p < 0.001$) as tested by ANOVA. **c)** Variance Partitioning Analysis (VPA). Environmental factors were the same as those used in CCA analysis. Microbial interactions included cohesion, total nodes, and total links. Sampled environmental factors included local temperature, local rainfall, and sampled piles. “Unexplained” indicates the variance that could not be explained by any one of these three groups. All analyses were carried out using the R packages “*vegan*”, “*ggprism*” and “*ggpubr*”.

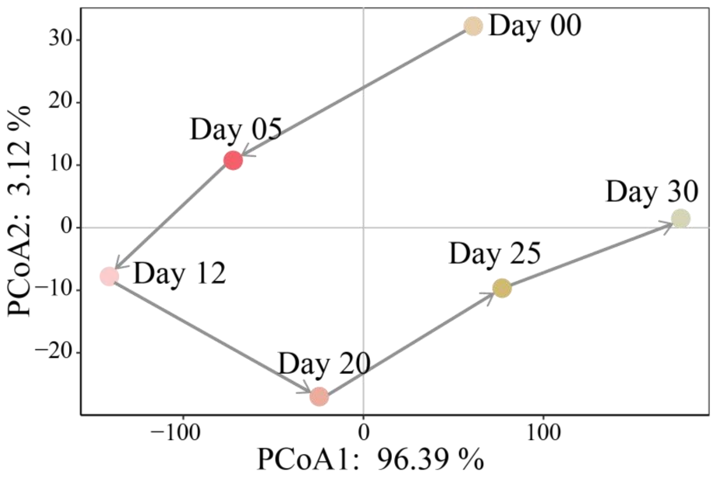

Fig. S8. Succession of the networks in response to the sampling time points. The construction method was same as previous described. Seventeen network topological parameters (calculated on the MENAP interface) were used for a principal co-ordinates analysis (PCoA) analysis to give an overview for the variation in the network at different sampling time points. The detailed values of these network topological parameters are shown in Supplementary Data 13. Arrows clarify the chronological order of the corresponding network.

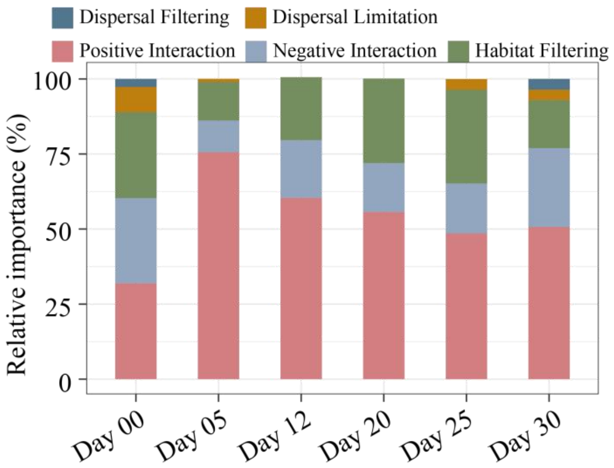

Fig. S9. Quantification of the relative contribution of community assembly processes to MEN links using an independent tool reported in Goberna et. al 2019. In detail, the process contained three processes: I) constructed the co-occurrence networks with set group, II) attribution of co-occurring patterns to assembly processes using spatial and environmental data, and III) calculation and analyzation of the phylogenetic distances between aggregated (co-occurring) or segregated (co-excluding) species.

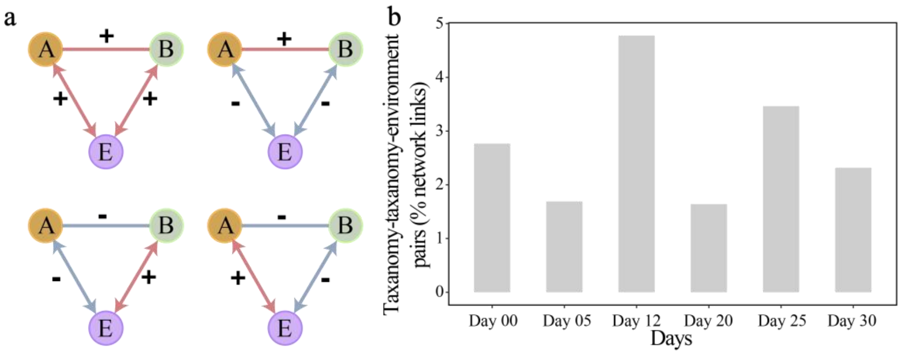

Fig. S10. Link Test for Environmental filtering (LTEF) for disentangling the contributions of environmental filtering to the observed network links. **a)** The framework to screen the potential taxon-taxon-environment links. A and B indicated the nodes (i.e., various taxa). E was the environmental factors with the same dimension as the taxa. Correlations between A-E as well as B-E were calculated based on the inter-domain network, which focused on the cross-domain associations between taxa and environment and eliminated links between taxa and taxa or environmental factor and environmental factor. Moreover, Both A-E and B-E should be significantly ($p<0.05$) correlated with correlation strengths above a set threshold $|r|$. **b)** The results of LTEF analysis.

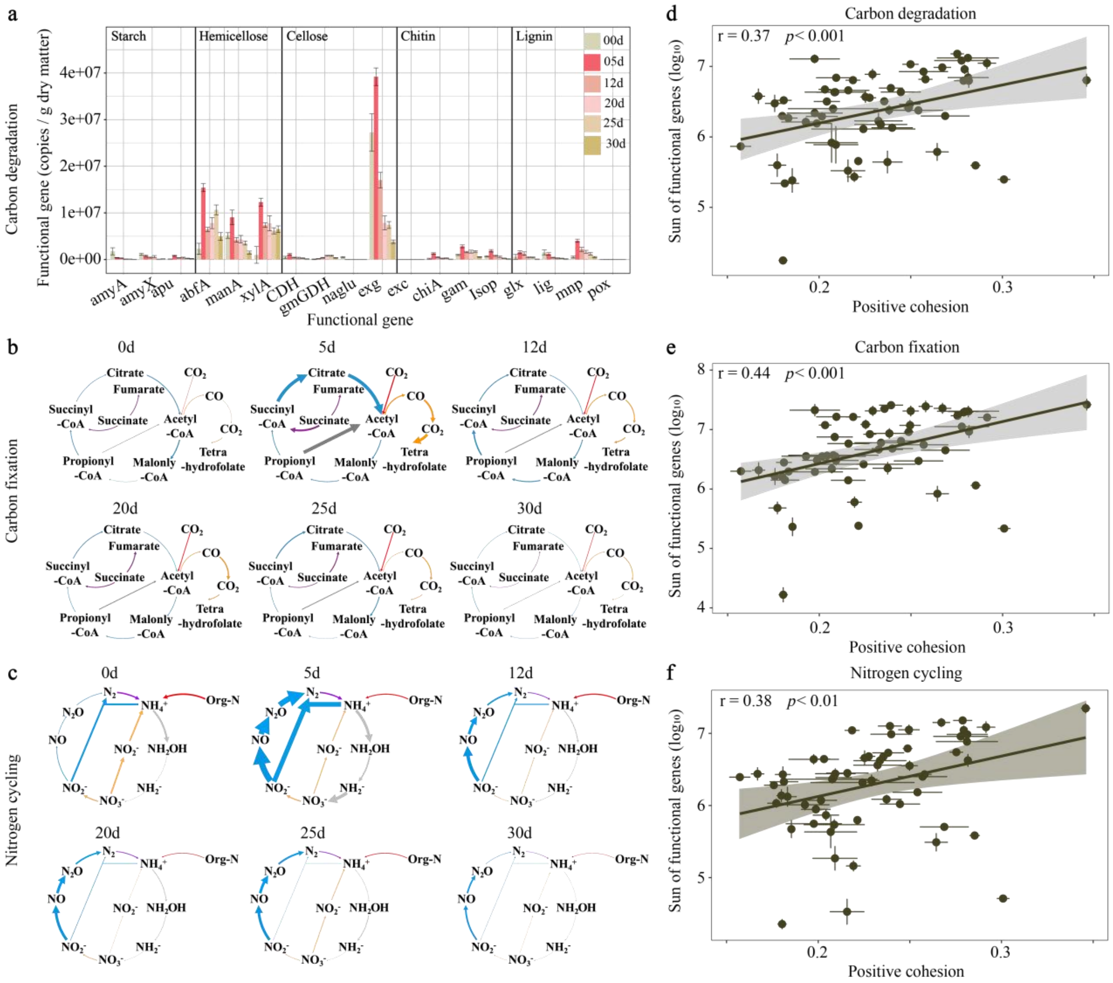

Fig. S11. Changes in functional genes related to carbon and nitrogen cycling. a) The copies of functional genes related to carbon degradation. b) The copies of functional genes related to carbon fixation. The arrows color indicate the different process in carbon fixation. c) The copies of functional genes related to nitrogen cycling. The arrows color indicate the different process in nitrogen cycling. d) The relationship between positive cohesion and the abundance of genes related to carbon degradation (n = 60 biologically independent samples). Data are presented as mean values +/- SEM. e) The relationship between positive cohesion and the abundance of genes related to carbon fixation (n = 60 biologically independent samples). Data are presented as mean values +/- SEM. f) The relationship between positive cohesion and the abundance of genes related to nitrogen cycling (n = 60 biologically independent samples). Data are presented as mean values +/- SEM.

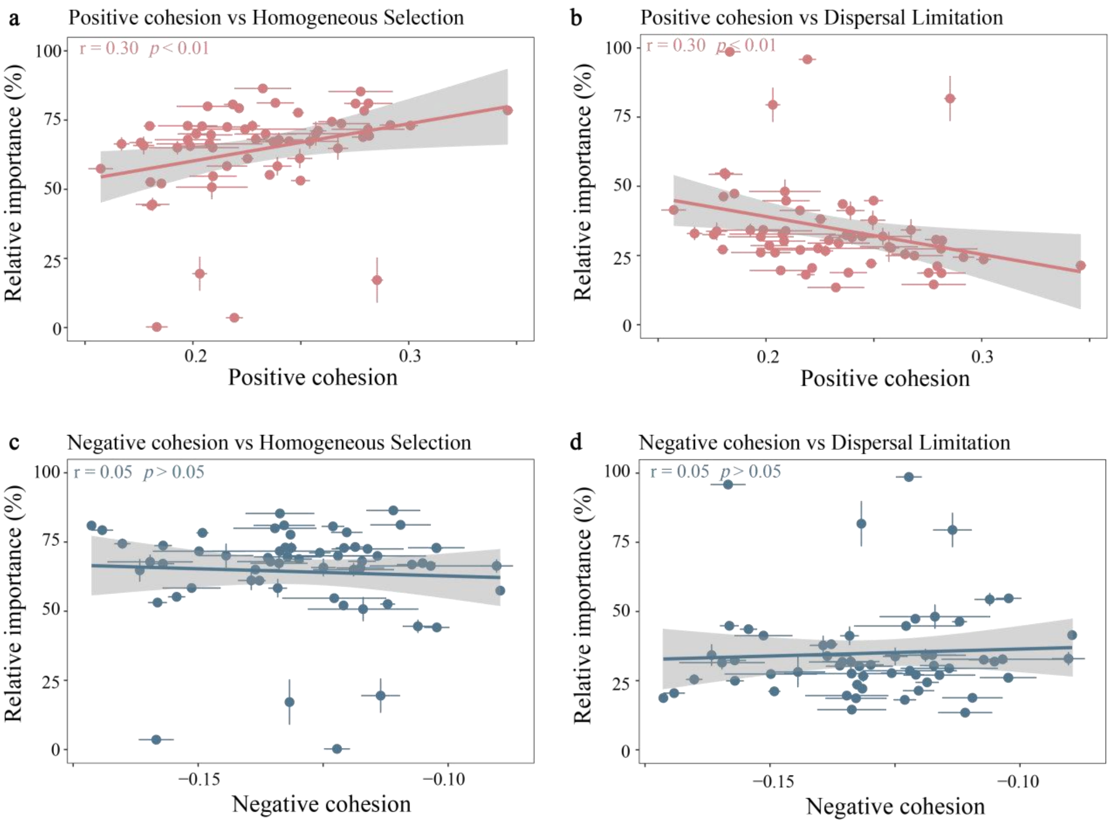

Fig. S12. Relationship between microbial interaction and community assembly. **a)** Relationship between positive cohesion and HoS. b**)** Relationship between positive cohesion and DL. **c)** Relationship between negative cohesion and HoS. d**)** Relationship between negative cohesion and DL. Correlation is calculated by Pearson. Grey shading denotes the 95% confidence intervals. Each sample is the average of three replicates. 60 biologically independent samples were included and data are presented as mean values +/- SEM.

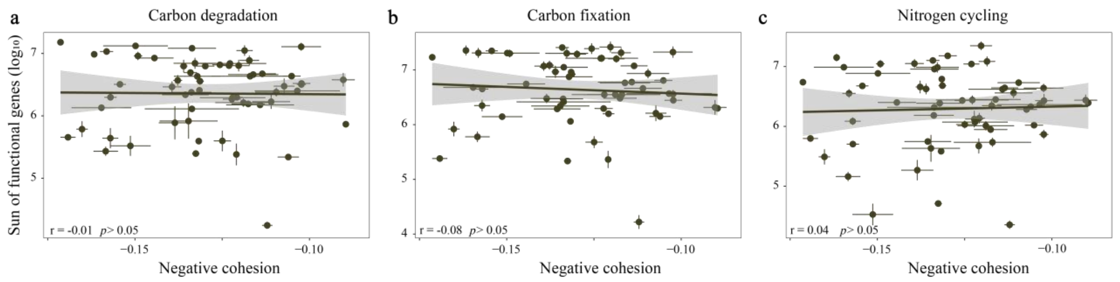

Fig. S13. Relationship between negative cohesion and functional potential. **a)** Carbon degradation. b) Carbon fixation. c) Nitrogen cycling. Grey shading denotes the 95% confidence intervals. Each sample is the average of three replicates. 60 biologically independent samples were included and data are presented as mean values +/- SEM.

Supplementary Note 3

Text S3.1 How positive interactions response to environment changes?

Random forest analysis The main environmental factors for regulating the variations of positive cohesion were identified by a classification random forest analysis. In this random forest model, environmental factors served as predictors for positive cohesion. To estimate the importance of these environmental factors, we used percentage increases in the MSE (mean squared error) of variables: higher MSE% values implied more important variables[10]. The significance of each predictor on the response variables was assessed with the "*rfPermute*" R package[11]. 500 decision trees were set, followed by 1000 random permutations and 5 repetitions of 10-fold cross-validation.

Mantel test We used Euclidean distances for environmental data and Bray-Curtis for positive interaction (positive cohesion, number of positive links and proportion of positive links). We further computed partial Mantel correlations between each positive interaction and environmental data with the R packages "*vegan*" and "*ecodist*"[12] (999 permutations).

Hierarchical partitioning analysis The hierarchical partitioning analysis were carried out with the R package "*hier.part*" to quantify the amount of variation in the proportion of positive cohesion, total positive links and proportion of positive links explained by individual environmental factors. Due to methodological constraints, we selected 12 environmental factors according to Fig. S14 and Fig. S15.

VPA analysis To provide a quantification of the relative contribution of different environmental factors to potential microbial cooperation (positive cohesion, total positive links, and proportion of positive links), we performed a VPA model via the R package "*vegan*". We calculated all possible combinations and the combination of the

list 4 factors was the optimal combination. Explanation rate of variance was calculated for univariate, bivariate, trivariate, and all four variables.

Linear regression analysis in Table S5 We used linear regression analysis (y = a + b×Temperature) to further confirm the relationship between temperature (independent variable) and positive cohesion, total positive links and proportion of positive links (dependent variable) in Table S5. The detailed structure is shown in Table S5. The linear regression analysis was carried out with the R package "*stats*". Temperature was always set as the independent variable. Positive cohesion, total positive links, and proportion of positive links were set as the dependent variable, respectively. "a" in the linear regression analysis refers to intercept, and "b" refers to slope.

Text S3.2 How positive cohesion response to temperature changes in each pile?

Linear regression analysis in Fig S17 To reveal the relationship between positive cohesion and temperature in each pile, we constructed another linear regression analysis (Positive cohesion = a + b×Temperature). Each linear model included samples only collected from this pile (6 sampling time points). Other details were similar as previously described in Text S3.1.

To further confirm the relationship between positive cohesion and temperature in each pile, we focused the temperature trajectories of individual communities and tested their relationship by linear regression analysis in each pile. The structure of linear regression analysis was the same as described in Test S3.1, where only the data for each pile were used (6 sample for each analysis).

The variation in temperature indicated that it needed 2.5 ± 0.8 days for the pile to warm up from the initial rise to over 50 °C and 7.4 ± 3.1 days to reduce from 50°C to below 40°C (Fig. S17a). Results of linear regression analysis showed that positive

cohesion could explain the change in temperature in nearly all the piles (R^2 = 0.55-0.92, $p < 0.05$), except for Pile C (Fig. S17b). Although no significant relationship was observed between positive cohesion and temperature in Pile C, it also achieved an R^2 of 0.3. Therefore, these results showed that the temperature variations in the different piles may be mainly caused by the variations in the positive cohesion.

Remarkably, an unexpected secondary warming occurred in Pile C. There is no doubt that the exclusion of data collected from pile C allows for better results. however, as Pile C completed the composting process, we did not exclude these data in order to respect the unexpected phenomena and exceptional samples.

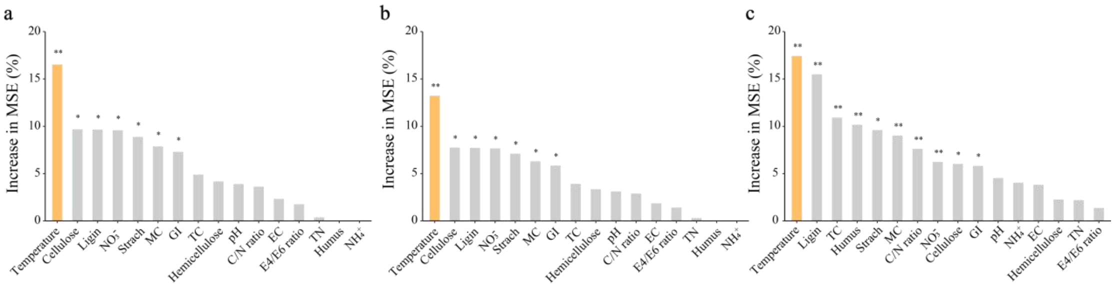

Fig. S14. Random forests analysis. **a)** Positive cohesion. **b)** Total number of positive links in microbial networks. **c)** Proportion of positive links in microbial networks.

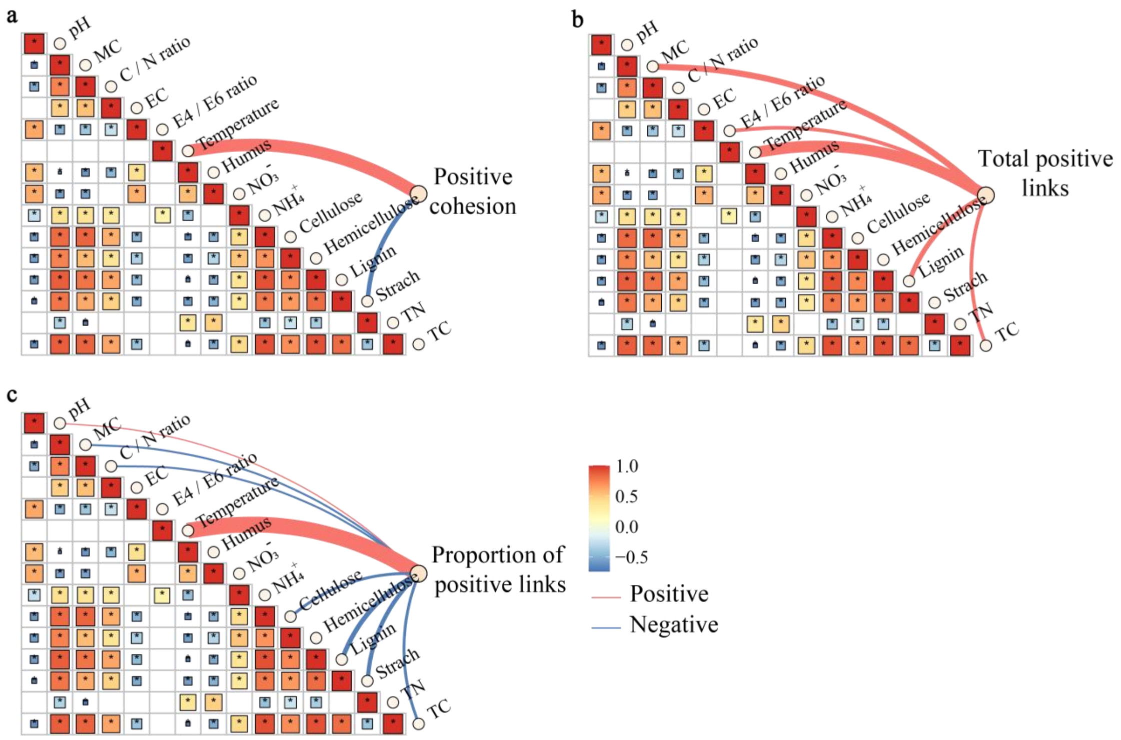

Fig. S15. Mantel tests. a) Relationship between environmental factors and positive cohesion. b) Relationship between environmental factors and total positive links. c) Relationship between environmental factors and proportion of positive links. Pairwise comparisons of environmental factors are shown in the triangle, with the colour gradient and square size denoting Pearson's correlation coefficient. The line size indicates the Mantel's r statistic between each environmental factor and positive cohesion, total positive links, and proportion of positive links. The color indicates positive (red) and negative (blue) correlation.

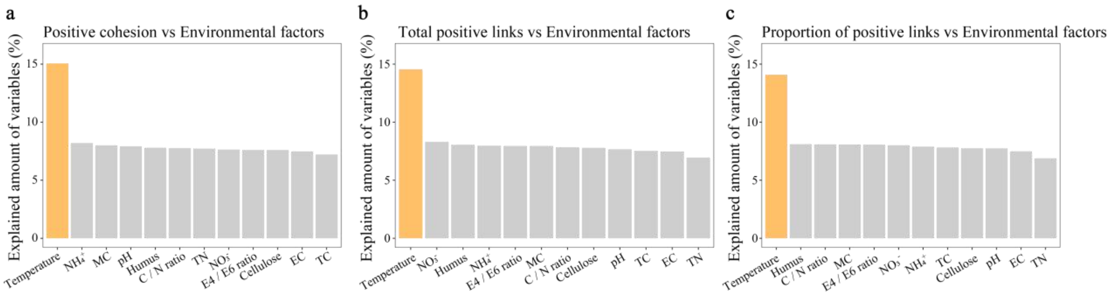

Fig. S16. Hierarchical partitioning analysis. **a)** The relationship between positive cohesion and environmental factors. **b)** The relationship between total positive links and environmental factors. **c)** The relationship between the proportion of positive links and environmental factors.

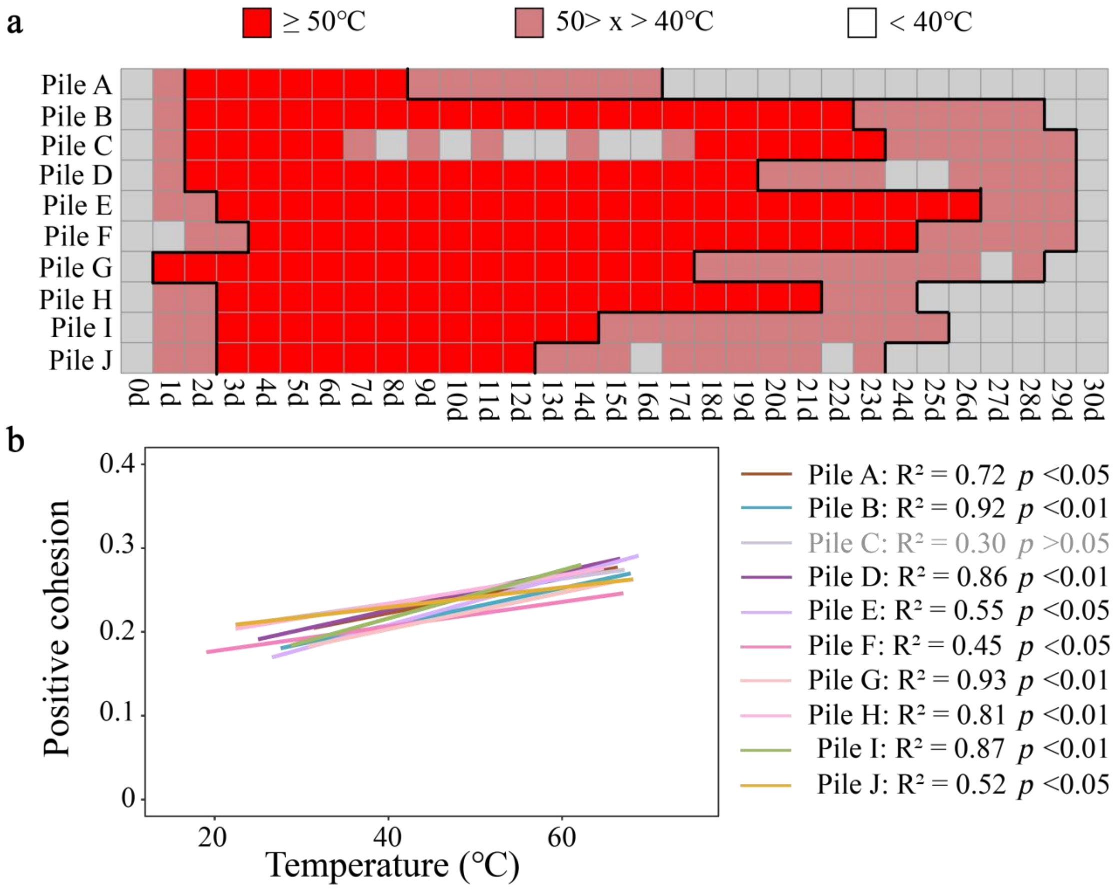

Fig. S17. Temperature in each pile. a) The temperature variations within different piles. The black line indicates the time required for the temperature transition. b) Linear model between positive cohesion (dependent variable) and temperature (independent variable) (n = 10 biologically independent samples for each pile).

Table S4 VPA analysis for the top 4 factors combinations to positive cohesion, total positive links, and proportion of positive links

Parameters	Composition	Variance explained rate					
Positive cohesion	Univariate	x1=pH	x2=Temperature	x3=Cellulose	x4=Lignin		
		0.0073	0.5277	0.0119	0.0133		
	Bivariate	x1+x2	x1+x3	x1+x4	x2+x3	x2+x4	x3+x4
		0.5299	0.0156	0.0178	0.5302	0.5332	0.0232
	Trivariate	x1+x2+x3	x1+x2+x4	x1+x3+x4	x2+x3+x4		
		0.5360	0.5381	0.0253	0.5318		
	Total	x1+x2+x3+x4					
		0.5407					
Total posotive links	Univariate	x1=NO_3^-	x2=Temperature	x3=Lignin	x4=TC		
		0.0793	0.7239	0.0038	0.0136		
	Bivariate	x1+x2	x1+x3	x1+x4	x2+x3	x2+x4	x3+x4
		0.7987	0.0802	0.0857	0.7249	0.7298	0.0158
	Trivariate	x1+x2+x3	x1+x2+x4	x1+x3+x4	x2+x3+x4		
		0.7957	0.8008	0.0898	0.7480		
	Total	x1+x2+x3+x4					
		0.8053					
Proportion of positive links	Univariate	x1=pH	x2=Temperature	x3=Lignin	x4=TN		
		0.0267	0.6063	0.0162	0.0164		
	Bivariate	x1+x2	x1+x3	x1+x4	x2+x3	x2+x4	x3+x4
		0.6349	0.0337	0.0298	0.6033	0.6640	0.0305
	Trivariate	x1+x2+x3	x1+x2+x4	x1+x3+x4	x2+x3+x4		
		0.6373	0.6766	0.0623	0.6601		
	Total	x1+x2+x3+x4					
		0.7053					

Table S5 The linear regression analysis for Temperature vs. positive cohesion, total positive links, and proportion of positive links. The adjusted R^2 and p value from linear regression is shown.

Formular	Fitted parameters	Value
Positive cohesion = a + b×Temperature	Intercept (a)	0.145781±0.010873
	Slope (b)	0.001854±0.000227
	R-squared	0.5278
	p value	< 0.001
Total positive links = c + d ×Temperature	Intercept (c)	-15.2343±13.8379
	Slope (d)	3.5984±0.2884
	R-squared	0.7239
	p value	< 0.001
Proportion of positive links = e + f×Temperature	Intercept (e)	0.466272±0.022603
	Slope (f)	0.004515±0.000471
	R-squared	0.6064
	p value	< 0.001

Supplementary Note 4

Text S4.1 Identification of ACT ASVs and their taxonomy

Only ASVs that fulfilled all four criteria could be defined as the ACT ASVs (Fig. S18 and S19). In detail, "overall abundant ASVs" focused on the mean relative abundance and should belong to the top 0.1% ASVs. In tested data, 155 ASVs could be identified as "overall abundant ASVs", accounting for 0.14% of total ASVs and 64.0% for the relative abundance (Fig. S20a). Meanwhile, 57 ASVs could be identified as "ubiquitous ASVs", which focused on the occurrence frequency (>80% samples). These "ubiquitous ASVs" accounted for 0.04% of total ASVs and 42.03% of their abundance (Fig. S20b). After that, "frequently abundant OTUs" were selected based on their relative abundances within a sample. 41 ASVs were selected, accounting for 0.05% of total ASVs and 45.78% for their abundance (Fig. S20c). Finally, due to the importance of "potential mutualism" in the composting process, we also used random forest analysis to obtain the ASVs which were highly associated with higher positive bacterial interaction. 17 ASVs were obtained, accounting for 0.02% percentage of ASVs and 16.6% of total abundance (Fig. S20d). Only three ASVs met all four criteria simultaneously (Fig. S20e), accounting for 0.002% of total ASVs and 12.3% of total abundance. In addition, the relative abundances of these ACT ASVs peaked on Day 05 and were significantly positively associated with temperature (Fig. S21). These results underlined the importance of ACT ASV as they had high relative abundance and frequency of occurrence and the potential to improve microbial cooperation.

Text S4.2 Keystone nodes

Defining keystone species or taxa, i.e., those with a high impact on the structure and functioning of ecosystems, is a critical, but difficult issue in ecology, especially in microbial communities. Based on the topological properties of networks, keystone nodes could be defined as module memberships. But such keystone nodes are not equivalent to keystone species or taxa typically used in ecology. Thus, in this study, we used keystone nodes instead of keystone species or taxa to refer to the topologically important nodes (ASVs) in the networks. For each node included in the constructed network, its Z_i value (i.e., within-module connectivity) and P_i value (i.e., among-module connectivity) were calculated to classify its topological role, including peripheral nodes ($Pi \leq 0.62$, $Zi \leq 2.5$), network nodes ($Zi \geq 2.5$, $Pi \geq 0.62$), connectors ($Zi < 2.5$, $Pi \geq 0.62$) and module nodes ($Zi \geq 2.5$, $Pi < 0.62$)[9,13,14]. All but the peripheral nodes were keystone nodes[9]. In our results, the keystone nodes included module nodes (i.e., nodes highly connected to other members in a module) and connectors (i.e., nodes linking different modules) (Fig. S22). We chose a 50% cutoff because the network reliability degraded significantly for OTUs containing >50% zeroes, and 50% cutoff was the recommended level[15]. Indeed, 50% OTU removal might exclude nondominant ASVs, but a false positive is likely more destructive[15]. In conclusion, all of the selected ACT ASVs were keystone nodes, which highlighted their importance.

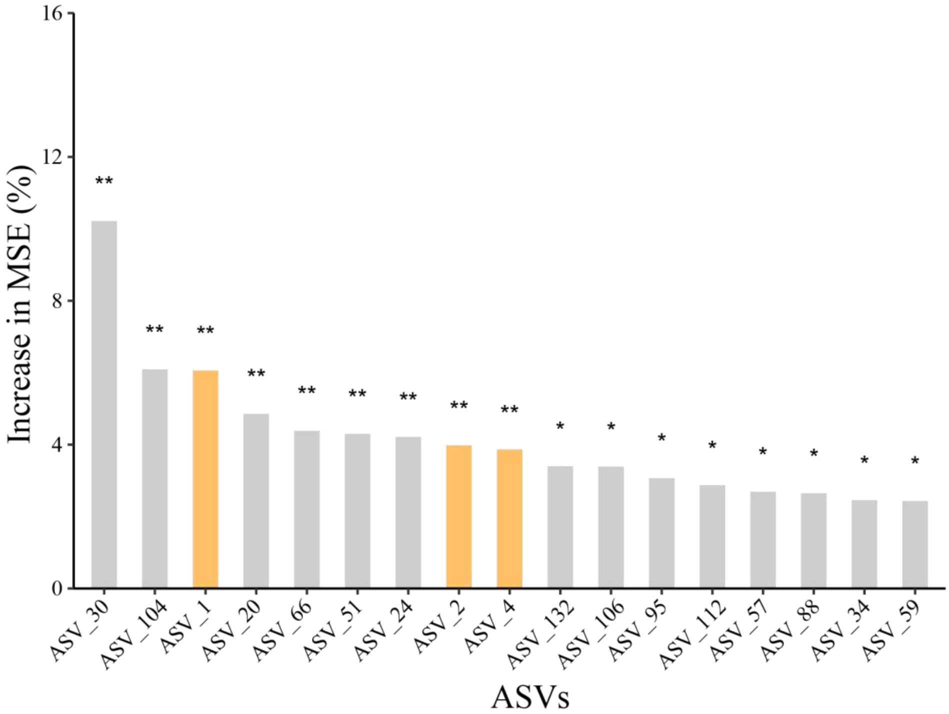

414 Fig. S18. Random forest model analysis to predict the ASVs with the greatest

415 importance to positive cohesion.

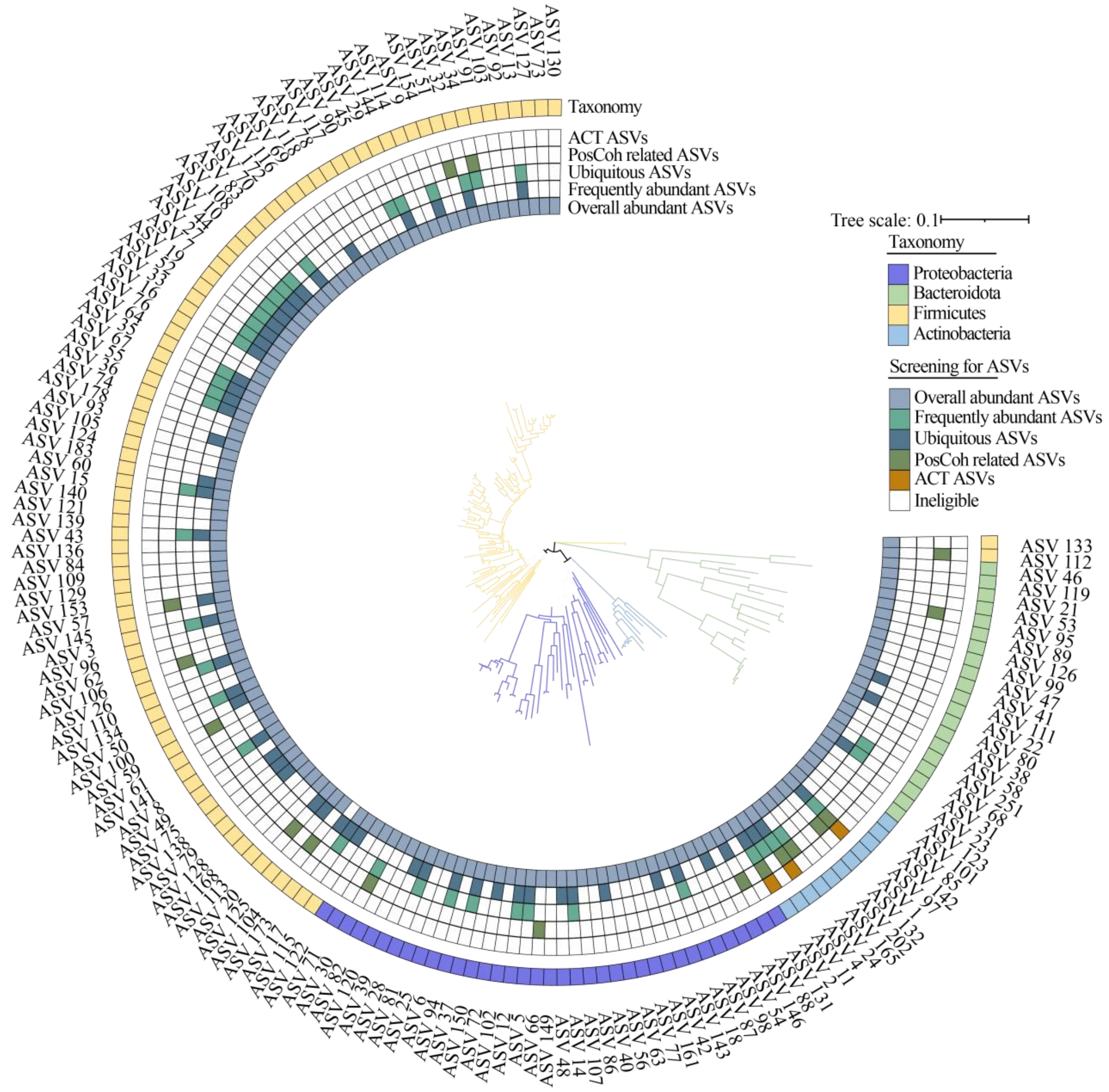

Fig. S19. Identification of ACT ASVs and phylogenetic analysis of the 16S rRNA gene (V4 region), including overall abundant ASVs, frequently abundant ASVs, ubiquitous ASVs, ASVs associated with positive cohesion (PC), and ACT ASVs.

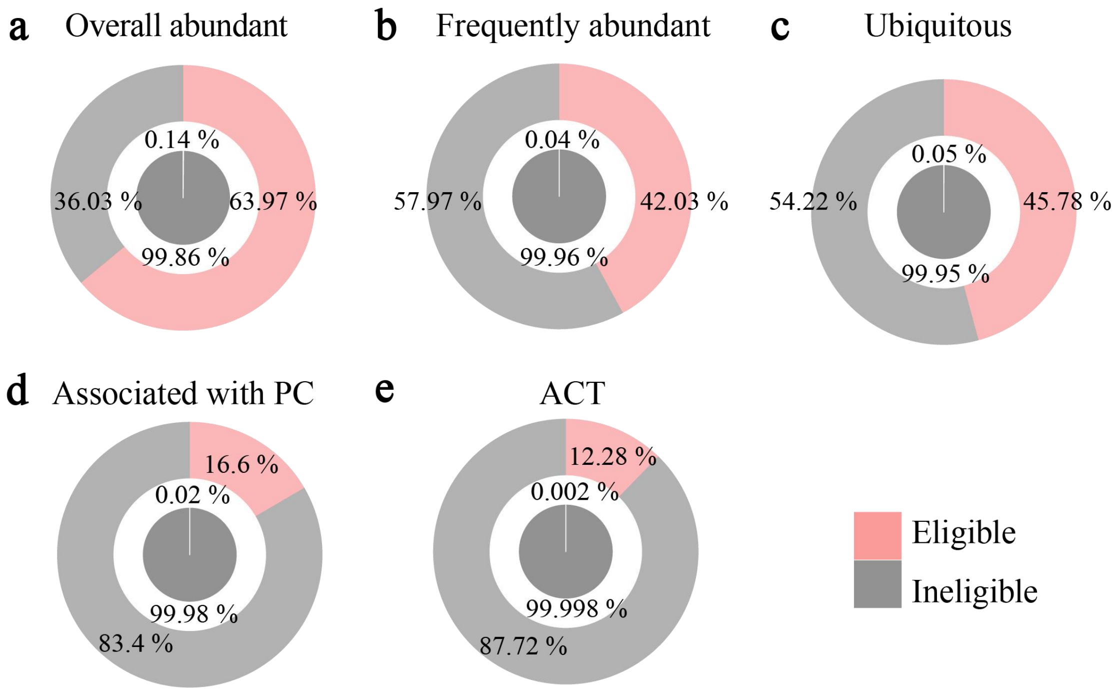

Fig. S20. Relative abundance and percentage of the composting ASVs. **a)** Overall abundant ASVs. **b)** Ubiquitous ASVs. **c)** Frequently abundant ASVs. **d)** ASVs associated with positive cohesion (PC). **e)** ACT ASVs (ASVs fulfilling all 4 conditions). The outer circle indicates the total relative abundance of ASVs. The inner circle indicates the percentage of ASVs. Eligible indicates ASVs that meet this screening criteria (e.g. only top 0.1% of mean relative abundance ASVs are considered to be “eligible” for overall abundance ASVs). Ineligible indicated ASVs that did not meet the screening criteria.

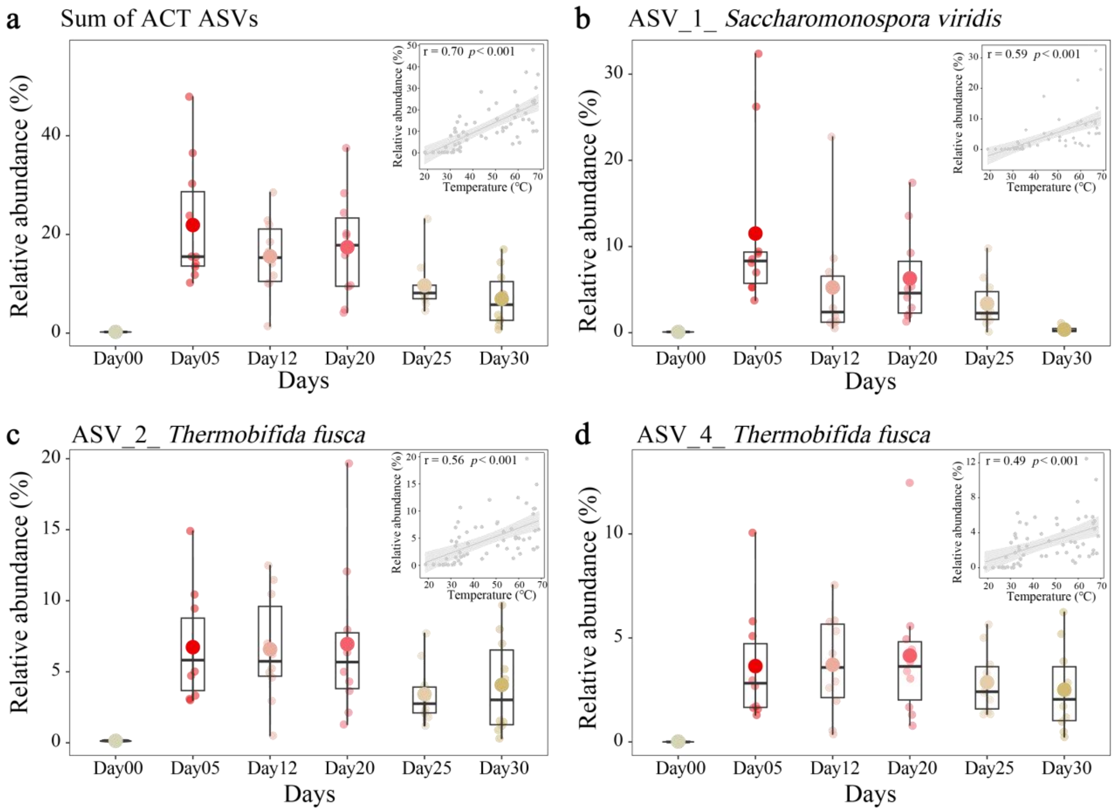

Fig. S21. Relative abundance of the ACT ASVs. **a)** Sum of ACT ASVs. **b)** Relative abundance of ASV_1. **c)** Relative abundance of ASV_2. **d)** Relative abundance of ASV_4. In the boxplots of panels, hinges indicate the 25th, 50th, and 75th percentiles, whiskers indicate 1.5 × interquartile ranges, and dots indicate values of individual samples (n = 10 biologically independent samples for each day). 60 biologically independent samples were included in all Pearson correlation analysis.

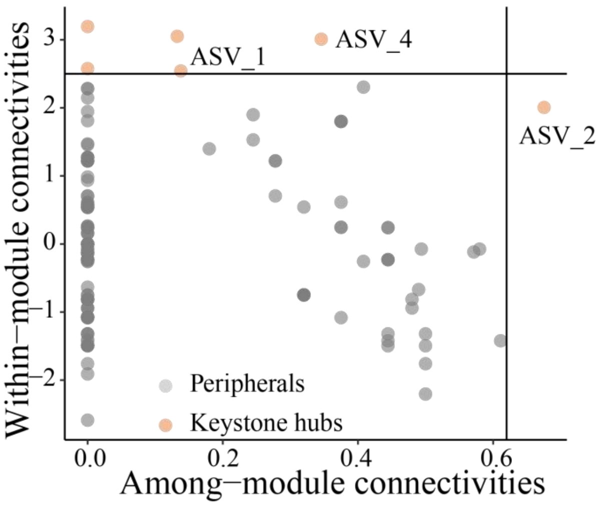

Fig. S22. Zi-Pi analysis for screening the keystone nodes.

Supplementary Note 5

Text S5 High temperature selection of slow-growing bacteria during composting

The ribosomal RNA operon copy number and mean copy number Since the maximum growth rate of a bacterium is proportional to the number of ribosomal RNA operon (rrn) copies it has, we calculated the rRNA copy number of different composting bacteria by matching ASVs to the Ribosomal RNA Operon Copy Number Database (rrndb) to infer the maximum growth rate of different composting bacteria[16]. The rrn copy number was matched the lowest available rank (e.g., if the species and genus levels are unclassified, then the family level would be the representative). Mean copy number (MCN) was calculated as the average rRNA copy number for each sample as described by Abreu et al. (2022). In detail, the MCN was calculated by weighting the rRNA copy number of each ASV and its relative abundance[17]. MCN for each sample is the average of three replicates.

Multiple linear regression model We conducted 2 multiple linear regression (MLG) models to reveal how temperature and nutrients affected MCN: (1) with all 60 samples, (2) with 50 samples (excluding samples collected on Day 00) to confirm the results. In both MLG models, temperature and nutrients were independent variables, and MCN was dependent variable. We used R packages "*tidyverse*" and "*leaps*" to construct the MLR and obtain R^2 for each model (Fig. S26a). Given the equal importance of optimal model and minimal model in the construction of MLG models, we further calculated the Bayesian Information Criterion (BIC) for the different models with a lower BIC indicating a better R^2.

Results of MLG model showed that although the R^2 for the combination of "temperature" and "TC" was not the largest among the 60 sample group, they had the smallest BIC and was the simplest model, therefore this MLR was the most

appropriate for the 60 sample group. Also, the combination of "temperature", "TC", “Cellulose”, and “Lignin” was both the simplest and most optimal model for the 50 samples group (Fig. S26 and Table S6). Temperature was the most important factor in both models, therefore, temperature was the key factor influencing the MCN. Considering the significantly negative correlation between temperature and MCN (Fig. S24), high temperature might therefore favor slow-growing bacteria.

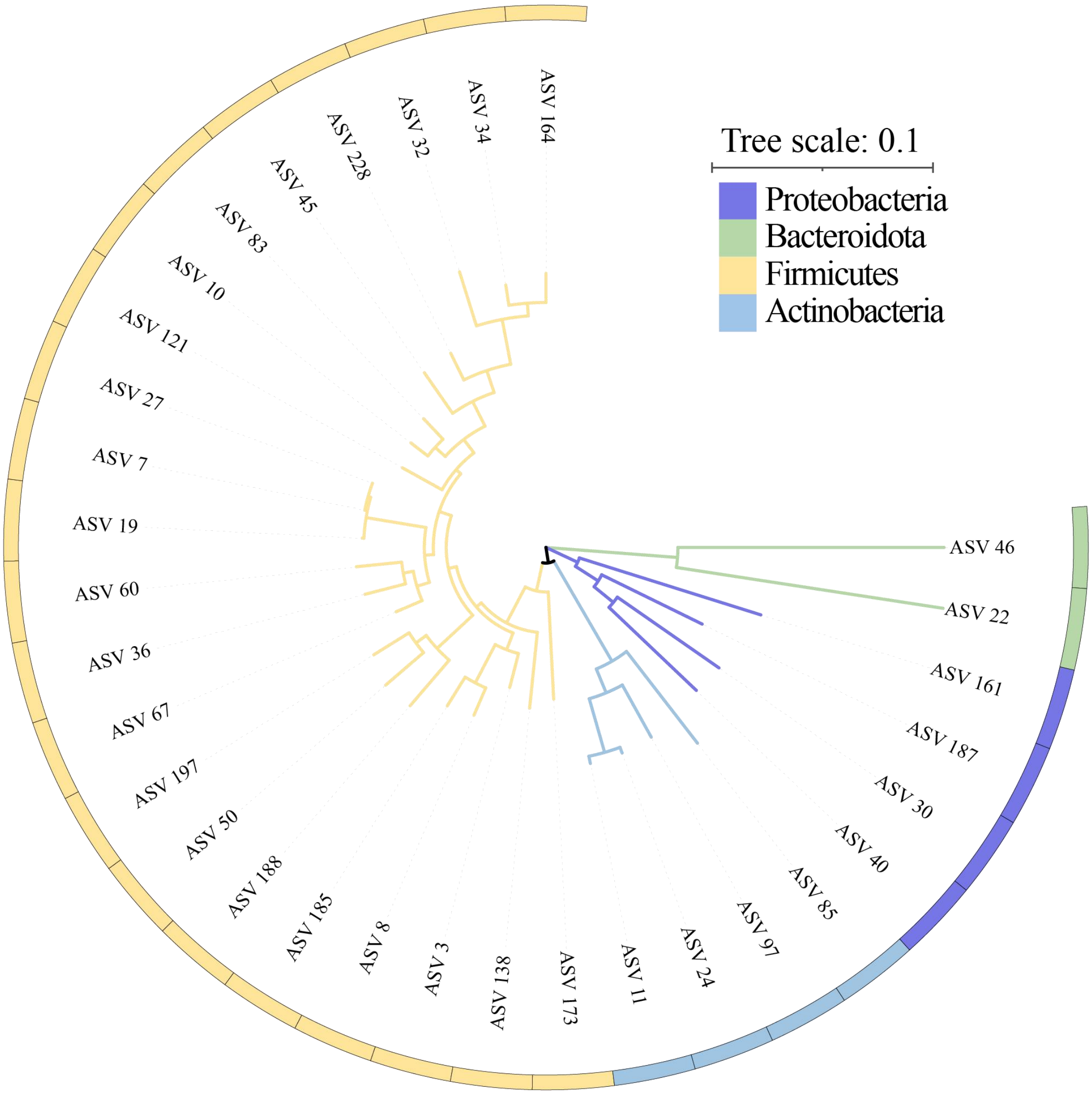

465 Fig. S23. Phylogenetic analysis of the 16S rRNA gene (V4 region) of

466 neighboring ASVs

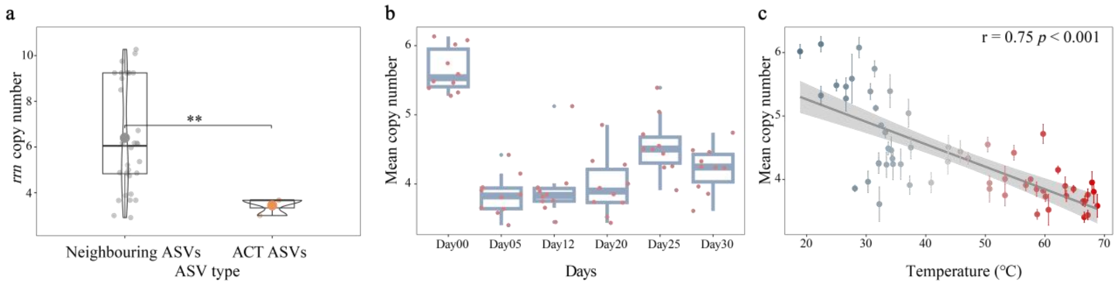

Fig. S24. Mean copy number (MCN) during the composting process. a) Ribosomal RNA operon copy number between neighbouring ASVs (n = 32) and ACT ASVs (n = 3). In the boxplots of panels, hinges indicate the 25th, 50th, and 75th percentiles, whiskers indicate 1.5 × interquartile ranges, and dots indicate values of individual samples. b) MCN for different day (n = 10 biologically independent samples for each day). c) The Pearson correlation between MCN and temperature (n = 60 biologically independent samples).

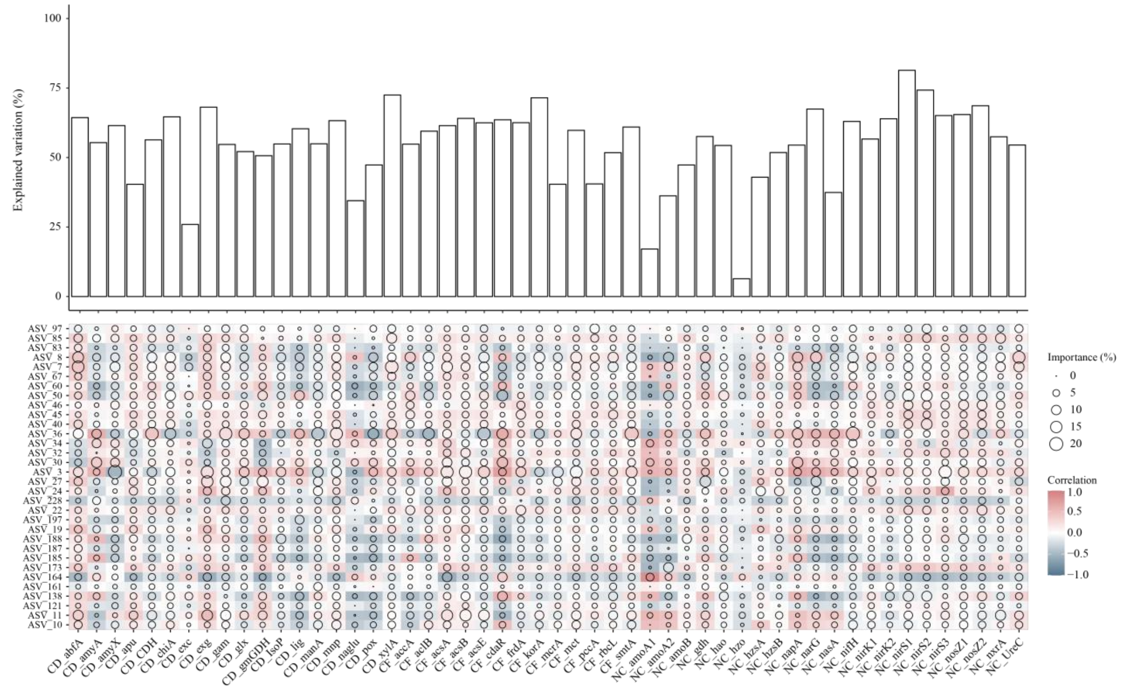

Fig. S25. Contributions of neighboring ASVs to composting ecological functions (carbon degradation, carbon fixation, and nitrogen cycling) based on correlation and random forest model. In this random forest model, the relative abundance of each neighboring ASV served as predictors for the copy number of each functional gene. The bar was the explained variation (i.e., %Var explained) calculated *via* R package "*randomForest*", indicating the degree to which all neighboring ASVs explained the variation in functional gene copy number. The circle size represents the variable importance of each neighboring ASV for the variation in functional gene copy number (i.e., percentage of increase of mean square error), which was calculated *via* R package "*rfPermute*". The heatmap is a Pearson's correlation between the relative abundance of each neighboring ASV and the copy number of each functional gene. The heatmap was constructed via the function "*cor*()". Colors represent Pearson's correlation coefficients.

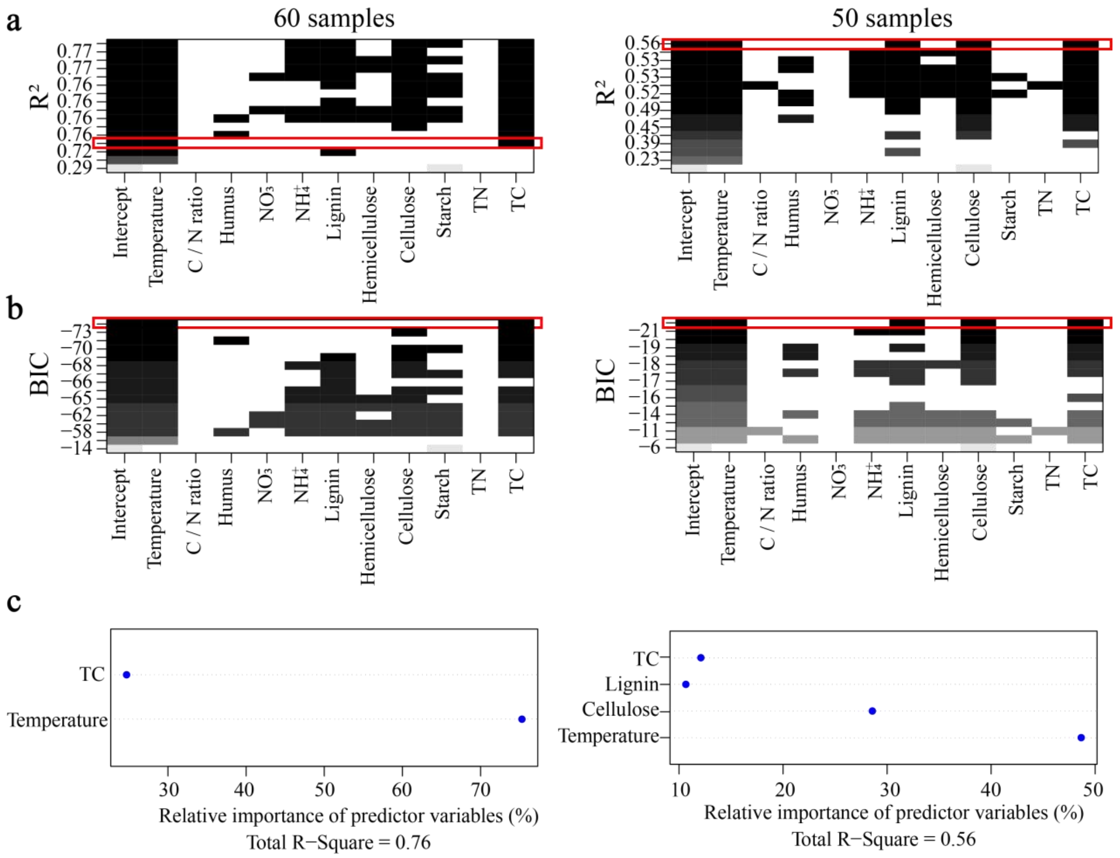

Fig. S26. Multiple linear regression between MCN and environmental factors. a) R^2 for different models. b) Bayesian Information Criterion (BIC) for different models. c) Relative importance of predictor variables. The red box indicates the selected model.

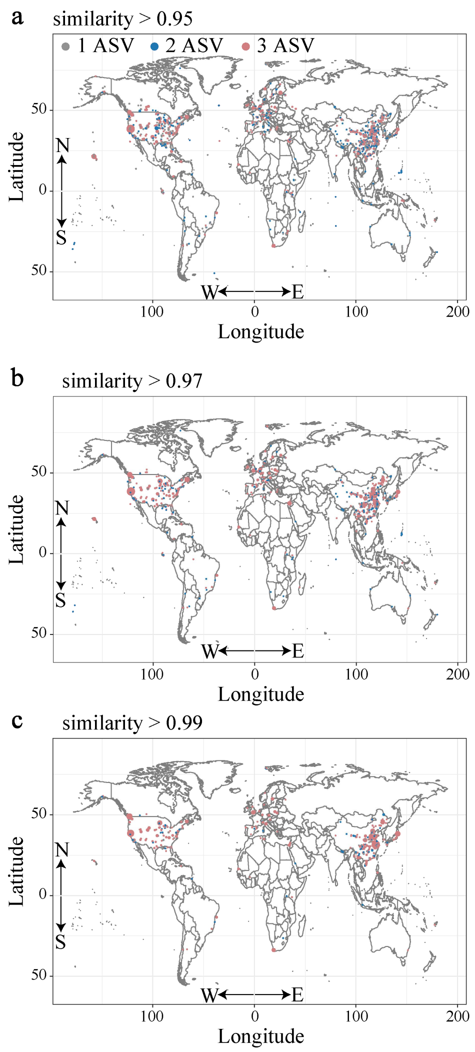

Fig. S27. Co-occurrence of the ACT ASVs. **a)** 95% similarity as a threshold. **b)** 97% similarity as a threshold. **c)** 99% similarity as a threshold. The co-presence of these ASVs was detected by searching the Sequence Read Archive (SRA) with the 16S rRNA gene sequences by IMNGS. Different colors were used to distinguish between the number of co-occurring ASVs. For example, blue dots indicated where 2 ASVs could be observed with a relative abundance of 0.1% in the same sample. Node size indicates the number of samples. Geographical locations and sample types of samples containing core species were extracted and visualised using the R packages "*sf*", "*rnaturalearth*" and "*rnaturalearthdata*".

Table S6 The detailed values of the constructed multiple linear regressions between temperature, nutrients and positive cohesion.

Samples number	Fitted parameters	Estimate	Std. Error	t value	Pr(>|t|)	Significant
Model including 60 samples	Intercept	4.8403	0.21806	22.20	2.0e-16	Yes
	Temperature	-0.0363	0.00304	-11.92	2.0e-16	Yes
	TC	0.0221	0.00316	6.98	3.4e-09	Yes
Model including 50 samples (excluding samples collected from day 0)	Intercept	5.3048	0.23823	22.27	2.0e-16	Yes
	Temperature	-0.0218	0.00499	-4.36	7.4e-05	Yes
	Cellulose	-0.3294	0.09060	-3.64	0.00071	Yes
	Lignin	0.2582	0.12234	2.11	0.04042	Yes
	TC	0.0222	0.00825	2.70	0.00985	Yes

Model including 60 samples: MCN=4.8403-0.0363*Temperature+0.0221*TC+ε
Model including 50 samples: MCN = 5.3048-0.0218*Temperature+0.0222*TC -0.3294*Cellulose+0.2582*Lignin+ε
ε is a constant in the multiple regression model
TC: Total Carbon

Supplementary Note 6

Text S6 Three main cobalamin-dependent enzymes

Cobalamin auxotrophy is the most common form of vitamin auxotrophy in marine plankton[18]. Six enzymes require cobalamin as a cofactor, and the central enzymes include *methionine synthase, metH, methylmalonyl-CoA mutase, mutA,* and *ribosomal small subunit methyltransferase, rsmB*, because methyl transfer and rearrangement reaction ae the main processes driven by cobalamin.

(1) *metH* and *rsmB*

Cobalamin-dependent methionine synthase catalyzes the transfer of a methyl group from N5-methyltetrahydrofolate to homocysteine, producing tetrahydrofolate and methionine[19]. Cobalamin-dependent methionine synthesis (*metH*) was present in at least 80% of the microbes, while Cobalamin-independent methionine synthesis (*metE*) was only present in 30%[19]. Moreover, most microbes can utilize the more efficient Cobalamin-dependent methionine synthesis pathway (*metH*) when Cobalamin is available, as opposed to the less efficient Cobalamin-independent pathway[19].

(2) *mutA*

This enzyme catalyzes the reversible isomerization of L-methylmalonyl-CoA to succinyl-CoA using 5’-deoxyadenosyl radical, produced by cobalamin, as a cofactor participating in the generation of radicals that allow isomerization of the substrate[19].

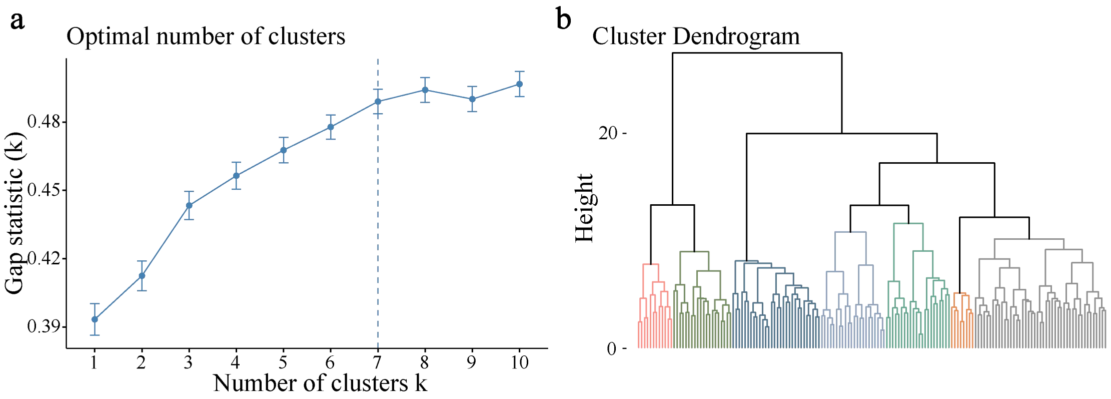

Fig. S28. Grouping of 159 MAGs based on the completeness of KEGG modules. **a)** Optimal number of clusters were predicted by using "*factoextra*" package in R. **b)** The detail for the seven groups.

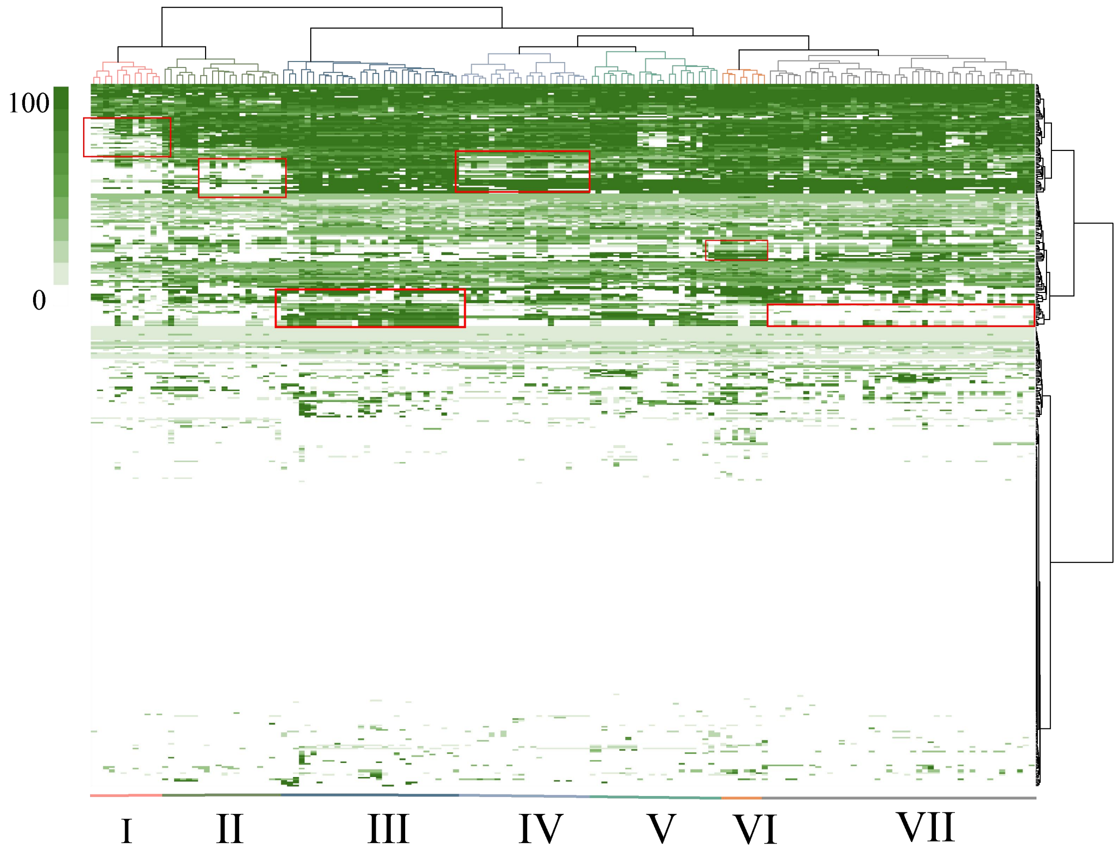

Fig. S29. Metabolic profiling of the bacterial community based on KEGG module completeness. Heatmap showing clustering of genomes (rows) by their KEGG module completeness (columns). Completeness ranges from 1 (green) to zero (white). Red rectangles are plotted on the heatmap to show differences between groups based on the completeness of the same module (each row) in different groups after clustering.

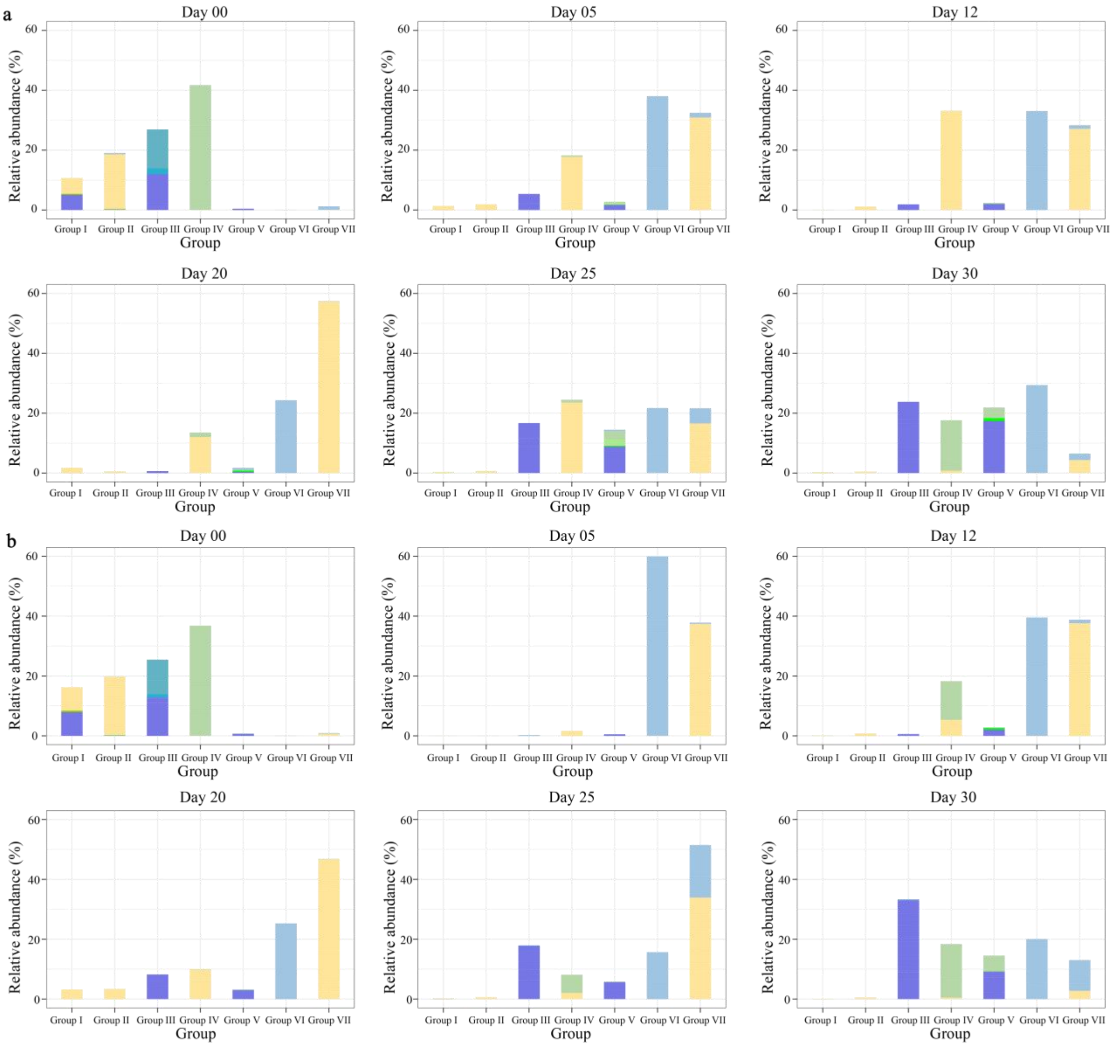

Fig. S30. The relative abundance and activity of the MAGs at different sample points. **a)** Relative abundance of MAGs. **b**) Relative activity of MAGs.

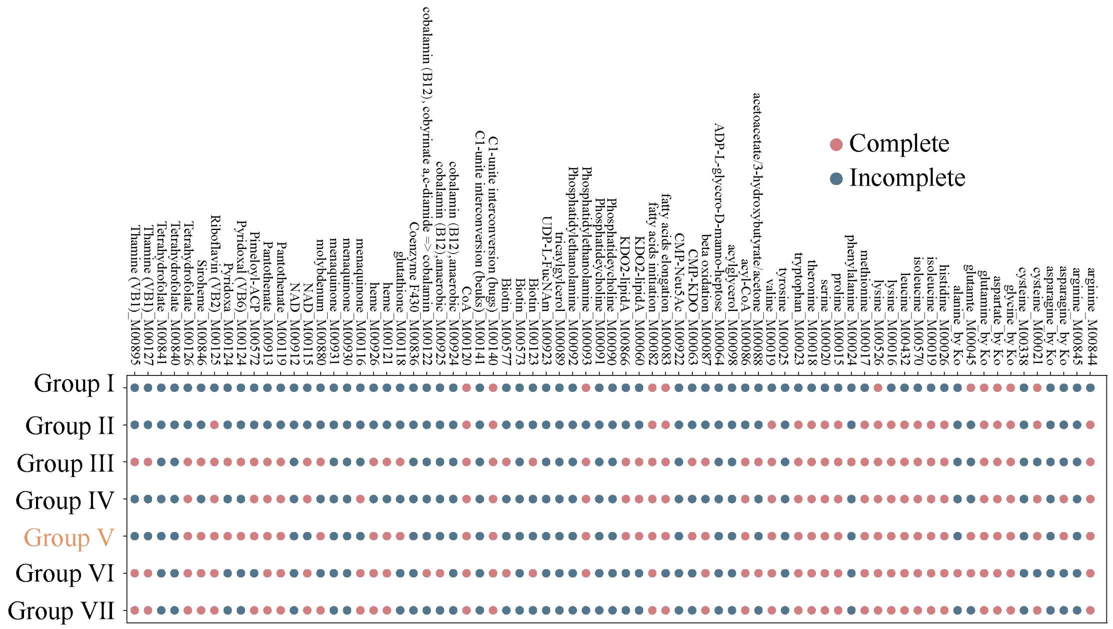

Fig. S31. Synthesis and transport of metabolites in Groups I–VII. The modules related to the amino acids (24 per), vitamins and cofactors (18 per), and lipids and fatty acids synthesis (28 per) were focused to reveal the synthetic potential. If module completeness was greater than 67% in over 50% of group members (MAGs), it could then be regarded as representative of the group. Red denotes >= 50% of the group are capable of synthesis/transport. Blue denotes < 50% of the group are capable of synthesis/transport.

Supplementary Note 7

Text S7.1 Consumption and accumulation of riboflavin

To confirm whether other metabolites showed the patterns observed for cobalamin, we utilized riboflavin as a control, as it is a biosynthetic vitamin like cobalamin (Fig. S35).

Experimental setup and parameters setting We cultured the same 10 Firmicutes strains in LB medium containing 1.5 ppm riboflavin. Meanwhile, we cultured Key and General group species in LB medium to reveal the accumulation of riboflavin. Samples were taken at 0, 12, 24, 48, 72h and centrifuged to obtain supernatants. Before detection, the supernatants were filtrated though a polyvinylidene fluoride membrane (0.22 μm) and diluted 50x to avoid signal suppression due to the matrix effect. After that, the supernatants were analyzed with an ultra-performance liquid chromatography-triple quadrupole mass spectrometer (XEVO TQ MS, Waters, UK). In detail, the ACQUITY UPLC HSS T3 column (1.7μm, 100 mm x 2.0 mm) (Waters, Manchester, UK) was used with a column temperature of 40 °C. Formic acid (0.1 % (v/v)) was used as solvent A and acetonitrile was used as solvent B. Gradient elution was achieved by solvent A: 0~1.0 min (90%~90%), 2.0~4.0 min (90%~50%), 4.0~5.0 min (50%~20%), 5.0~6.0 min (20%~20%) and 6.0~7.0 min (20%~90%). The injection volume was 10.0 μL and the flow rate was 0.2 mL min^{-1}. Only characteristic ions satisfying all the setting parameters were considered as the test cobalamin (Table S14).

The consumption of riboflavin in Firmicutes showed a trend of decrease and then increase (Fig. S35a), which was a different pattern from that of cobalamin. This result indicated that Firmicutes could secrete riboflavin extracellularly. Different trends were also observed in the accumulation of riboflavin and cobalamin. The

accumulation of riboflavin was observed for all the tested species, rather than being secreted only by the key group (Fig. S35b). Riboflavin accumulation showed an increasing trend in all groups, unlike cobalamin, which could only be secreted by the key group.

Text S7.2 Comparative genomic analysis

Extraction of genome DNA Genomic DNA for *Thermobifida fusca* and *Saccharomonospora viridis* were extracted with the GenElute™ Bacterial Genomic DNA Kit Protocol (NA2100, SIGMA, Japan), following the manufacturer's instructions. DNA concentration and purity were measured using Qubit 3.0 (Thermo Fisher Scientific, Waltham, USA) and Nanodrop One (Thermo Fisher Scientific, Waltham, USA) at the same time.

Library preparation and sequencing Sequencing libraries were generated using the ALFA-SEQ DNA Library Prep Kit. The library quality was assessed on the Qubit 4.0 Fluorometer (Life Technologies, Grand Island, NY) and Qsep400 High-Throughput Nucleic Acid Protein Analysis system (Houze Biological Technology Co, Hangzhou, China). Finally, the library was sequenced on an Illumina Novaseq6000 platform (MAGIGENE Biological Technology Co. Ltd Guangzhou, China). and 150 bp paired-end reads were generated.

Data preprocessing The raw data were quality controlled, and the high-quality sequences were used for downstream data analysis. Specific processing steps are as follows: (1) Remove low mass bases at both ends of reads (mass value <20), and remove short reads (default setting was 50bp); (2) Remove reads with a specific number of N bases (default setting was 10bp); (3) Remove reads between overlap exceeding a certain threshold (default set as 15bp) and Adapter; (4) Remove duplication pollution;

De novo Genome Assembly The reads with low sequencing quality values were filtered by quality control steps, and the clean data after data filtering and quality control were de novo assembled using SPAdes v3.13.0 to obtain high-quality contigs fragments.

Comparative genomics analysis Comparative genomics analysis were carried out between *Thermobifida fusca* and *Saccharomonospora viridis* and MAG. 73 and MAG. 406, respectively. The details are as follow: (1) Average nucleotide identity (ANI) is one of the most powerful measures to identify the kinship distance of bacterial genomes. When ANI > 95% it indicates that two genomes belong to the same species. (2) Collinearity between the genomes was identified by performing an alignment between the target genome and the reference genome, using MUMmer software (Version 3.23).

Results showed that the ANI between MAG 73 and *Thermobifida fusca* was 99.8%, and MAG 406 and *Saccharomonospora viridis* was 99.6% (Fig. S37 and Table S8-S9). Moreover, KEGG analysis confirmed that biosynthesis pathway of cobalamin in the two purchased strains was also consistent with that of MAG 73 and MAG 406 (Fig. S37c). Overall, these results indicated that two purchased strains were consistent with MAG 73 and MAG 406.

Text S7.3 Medium

LB-Lennox medium (per litre): peptones 10 g, yeast extract, 5 g, NaCl 5g

De Man Rogosa Sharpe (MRS) medium: Peptone 10.0 g, Beef dip powder 5.0 g, Yeast Extract 4.0 g, Glucose 20.0 g, Dipotassium hydrogen phosphate 2.0 g, Triammonium citrate 2.0 g, Sodium acetate 5.0 g, Magnesium sulphate 0.2 g, Manganese sulphate 0.05 g, Tween 80 1 ml.

2216E medium: Peptone 5.0 g, Yeast Extract 1.0 g, Ferric Citrate 0.1 g, Sodium Chloride 19.45 g, Magnesium Chloride 5.9 g, Magnesium Sulfate 3.24 g, Calcium Chloride 1.8 g, Potassium Chloride 0.55 g, Sodium Bicarbonate 0.16 g, Potassium Bromide 0.08 g, Strontium Chloride 34.0 mg, Boric Acid 22.0 mg, Sodium Silicate 4.0 mg, Sodium Fluoride 2.4 mg, Ammonium Nitrate 1.6 mg, Disodium Phosphate 8.0 mg

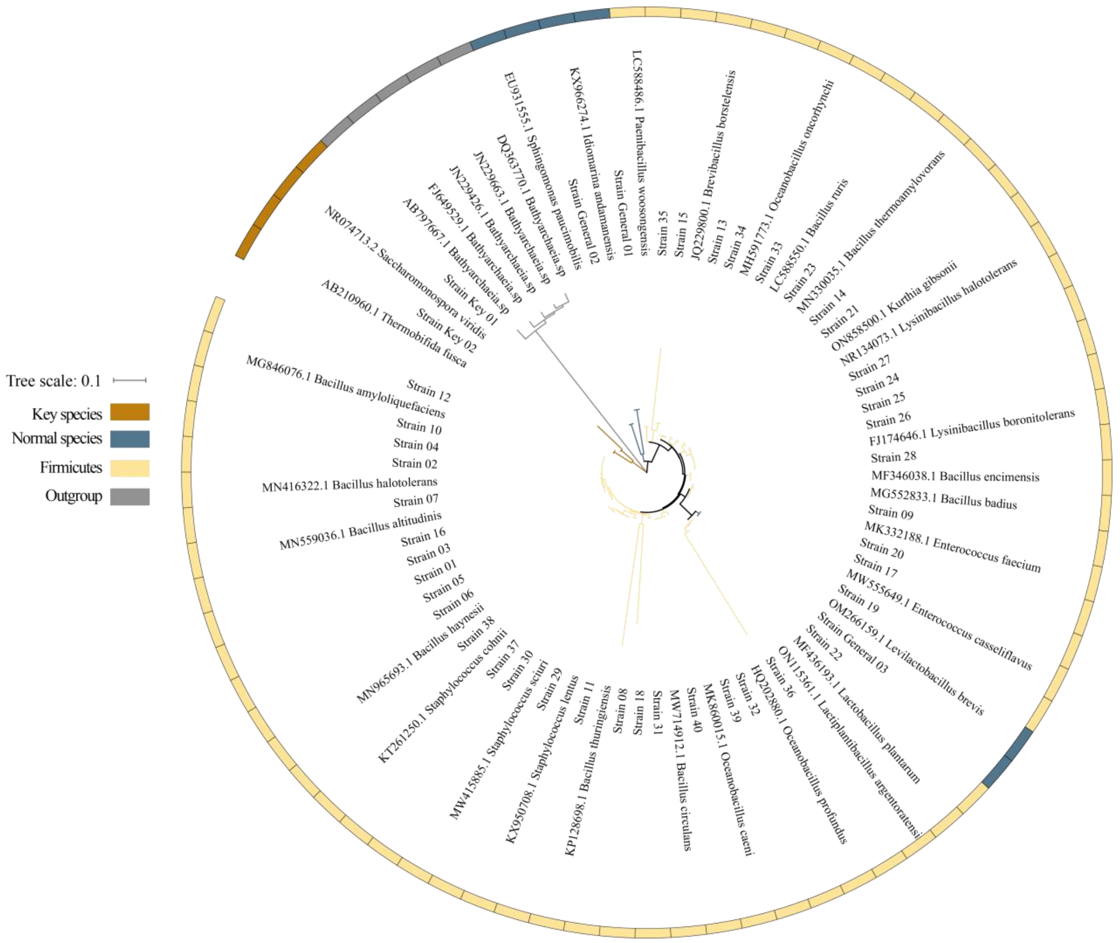

Fig. S32. Phylogenetic analysis of the 16S rRNA gene (whole length) of the tested strains. 40 strains affiliated with Firmicutes, and 3 general species (*Sphingomonas paucimobilis, Lactobacillus brevis* and *Idiomarina andamanensis*) were isolated from compost. *Thermobifida fusca* and *Saccharomonospora viridis* were purchased from the Deutsche Sammlung von Mikroorganismen und Zellkulturen.

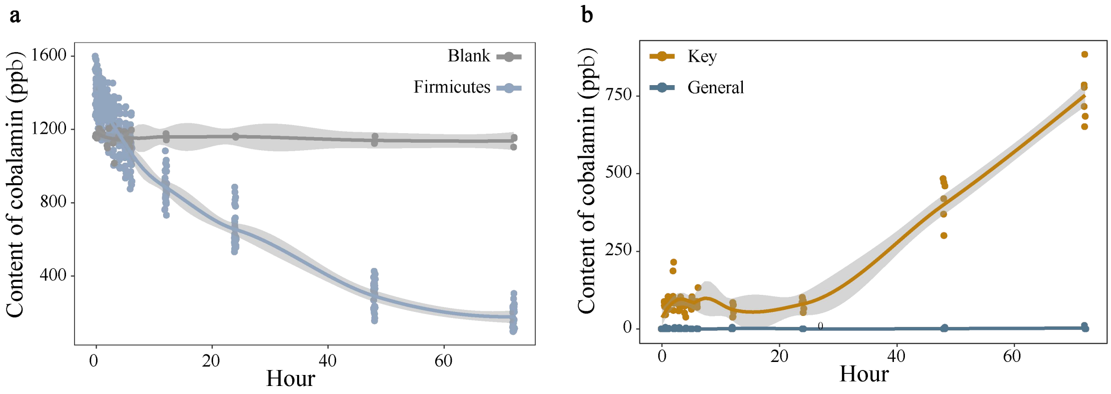

Fig. S33. Consumption and accumulation of cobalamin. a) The consumption of cobalamin in Firmicutes. b) The accumulation of cobalamin in the Key group species and the General group. Grey shaded areas are 95% confidence intervals.

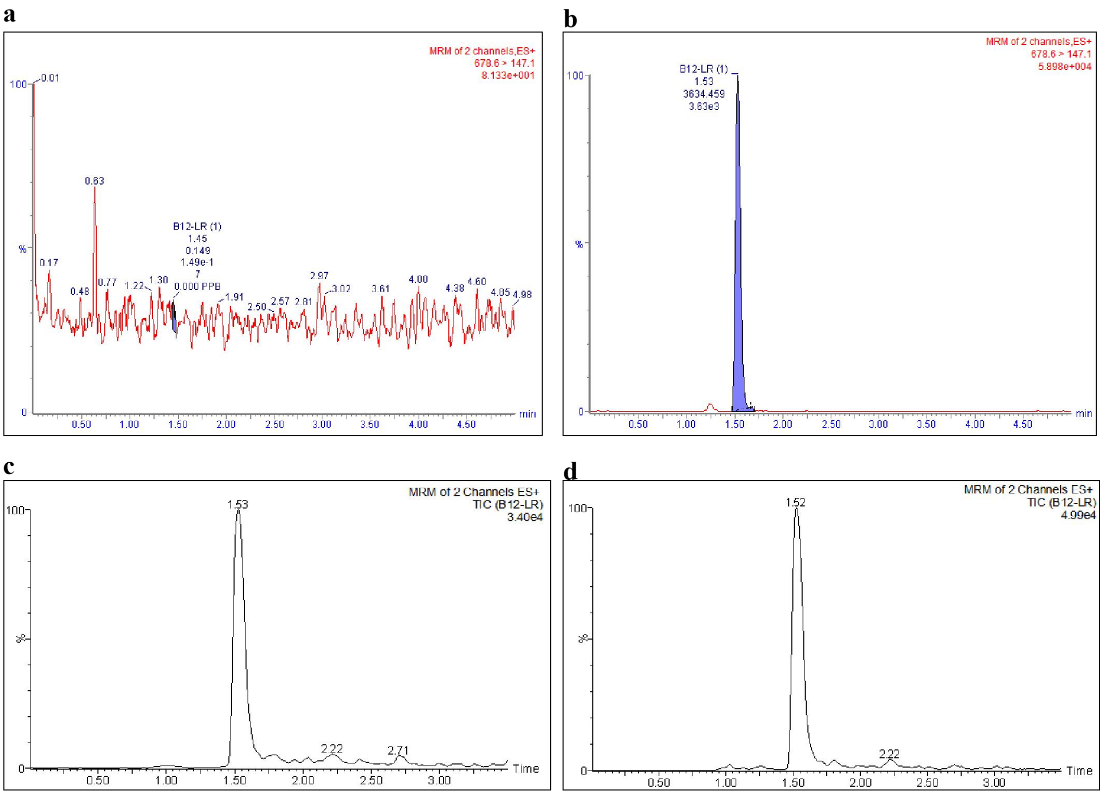

Fig. S34. The testing for benzimidazolyl cobamides. **a)** Without benzimidazolyl cobamides. **b)** Adding 20ppm benzimidazolyl cobamides. **c)** The medium after culturing *Thermobifida fusca* for 72h (diluted 50x). **d)** The medium after culturing *Saccharomonospora viridis* for 72h (diluted 50x).

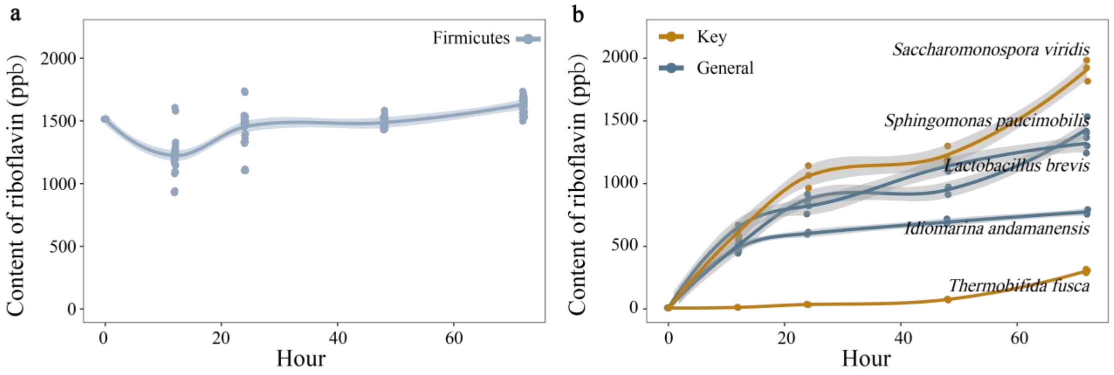

Fig. S35. Consumption and accumulation of riboflavin. a) The consumption of riboflavin in Firmicutes. b) The accumulation of riboflavin in species of the Key and General groups. Grey shaded areas denote the 95% confidence intervals.

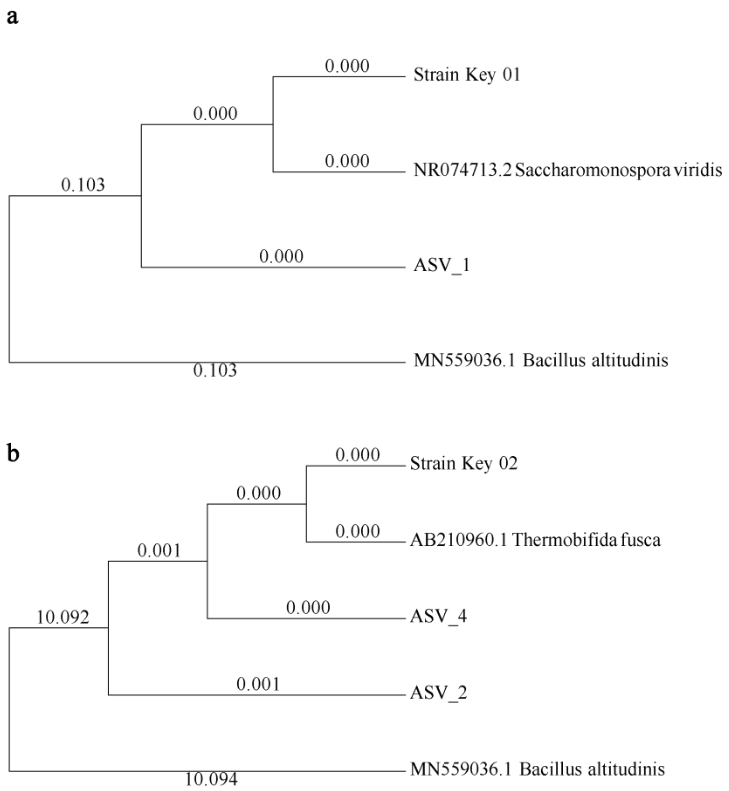

Fig. S36. Phylogenetic analysis of the 16S rRNA gene of the purchased strains (whole length) and the obtained ACT ASVs. **a)** *Saccharomonospora viridis* and ASV_1. **b)** *Thermobifida fusca*, ASV_2 and ASV_4. The number above the branch indicates the branch lengths.

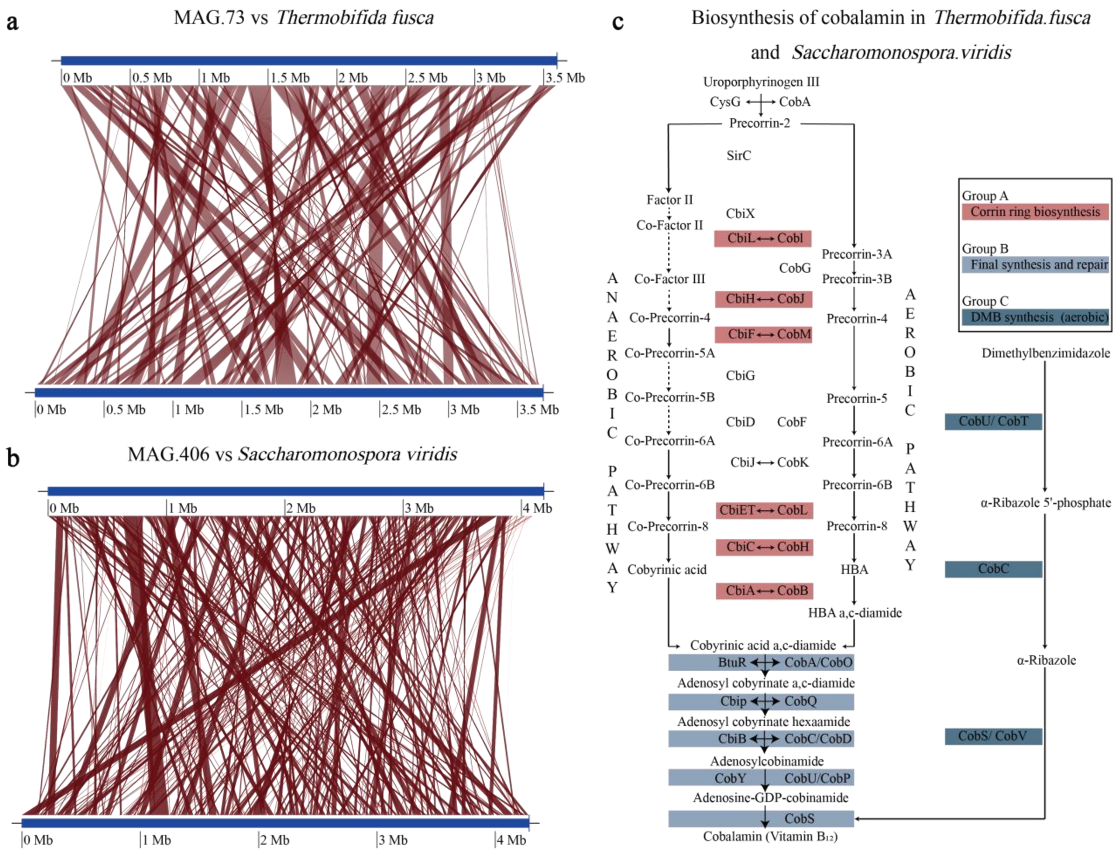

Fig. S37. Comparative genomic analysis between purchased strains and MAGs. a) Comparative genomic analysis between MAG. 73 and *Thermobifida fusca*. Upper line represents the genome of MAG. 73 and lower line represents *Thermobifida fusca.* b) Comparative genomic analysis between MAG. 406 and *Saccharomonospora viridis*. Upper line represents the genome of MAG. 406 and lower line represents *Saccharomonospora viridis.* c) The biosynthesis pathway of cobalamin in the purchased strains.

Table S7 PCR reaction system for high-throughput sequencing

Reagent	Usage
2x Premix Taq	25 μL
PCR Reverse Primer (10 μmol/L)	2 μL
cDNA	1.0 μL
RNase Free H_2O	50 μL
Initial denaturation: 94℃, 5min	
30 cycles: 94℃, 30s; 52℃, 30s; 72℃, 30s	
Final elongation:72℃, 10min	

Table S8 The genomic details of the purchased strains

Strain	Total_length (bp)	Mean_length (bp)	N50 (bp)	N90 (bp)	GC (%)
Thermobifida fusca	3685329	89886.07	204614	75707	67.47
Saccharomonospora viridis	4285697	389608.82	973787	435231	67.35

Table S9 Comparative genomes between purchased strains and MAGs

Strain	Reference MAG	ANI (%)	Mapped fragment	Query fragment
Thermobifida fusca	MAG. 73	99.8	1103	1153
Saccharomonospora viridis	MAG. 406	99.6	1201	1257

Table S10 Experiment Design of the effect of supernatants and cobalamin on the growth of Firmicutes

Medium	Temperature	Groups	Detail
LB medium	37 ℃ and 50 ℃	Key group	10% (v/v) supernatants of *Thermobifida fusca**
			10% (v/v) supernatants of *Saccharomonospora viridis*
		General group	10% (v/v) supernatants of *Sphingomonas paucimobilis*
			10% (v/v) supernatants of *Lactobacillus brevis*
			10% (v/v) supernatants of *Idiomarina andamanensis*
		Cobalamin Group	10% (v/v) cobalamin (1.5 ppm)
		Control	additional 10% (v/v) LB medium (with 1 ppm Co)

* The supernatants were collected from targeted strains after culturing for 72 hours in LB medium with 1 ppm Co (2.188 mg / L $CoCl_2$).

Table S11 Specific primers[20] and qPCR reaction system

Primers	Sequences	Target
γ-proteobacteria-PF	TCGTCAGCTCGTGTYGTGA	Proteobacteria
γ-proteobacteria -PR	CGTAAGGGCCATGATG	
BacteroideteF	CRAACAGGATTAGATACCCT	Bacteroidota
BacteroideteR	GGTAAGGTTCCTCGCGTAT	
FirmicutesF	GAAACTYAAAGGAATTGACG	Firmicutes
FirmicutesR	ACCATGCACCACCTGTC	
ActinobacteiaF	TACGGCCGCAAGGCTA	Actinobacteria
ActinobacteiaR	CRTCCCCACCTTCCTCCG	
BrevF	CAGTTAACTTTTGCGAGTCAGCAG	*Lactobacillus*
BrevR	CGTCAGGTTCCCCACATAACTC	*brevis*

Reaction system:

PCR Forward Primer (10 μmol/L) 0.4 μL
PCR Reverse Primer (10 μmol/L) 0.4 μL
cDNA 1.0 μL
TB Green Premix Ex Taq II 10.0 μL
RNase Free H_2O 8.2 μL
Initial denaturation: 95°C, 30s
45 cycles: 95°C, 5s and 60°C, 30s
Primer annealing:95°C, 15s; 60°C, 60s and 95°C, 15s

Table S12 Experiment Design of Co-culture between Firmicutes and other species

Medium	Temperature	Groups	Co-culture
LB medium and CL medium*	37 ℃ and 50 ℃	Key group	40 Firmicutes strains + *Thermobifida fusca*
			40 Firmicutes strains + *Saccharomonospora viridis*
		General group	40 Firmicutes strains + *Sphingomonas paucimobilis*
			40 Firmicutes strains + *Lactobacillus brevis*
			40 Firmicutes strains + *Idiomarina andamanensis*
		Control	40 Firmicutes strains

* CL medium was the composting leachate collected from the compost.

Table S13 Experiment Design of the absorption (Firmicutes) and synthesis (Key group) of cobalamin

Medium	Temperature	Groups	Co-culture
LB medium + 1.0 ppm Co	37 °C	Key group	*Thermobifida fusca*<br>*Saccharomonospora viridis*
LB medium + 1.0 ppm Co		General group	*Sphingomonas paucimobilis*<br>*Lactobacillus brevis*<br>*Idiomarina andamanensis*
LB medium + 1.5 ppm cobalamin		Firmicutes	10 Firmicutes strains

* 1 ppm Co was added by 2.188 mg / L $CoCl_2$.

Table S14 Detail of characteristic ions, cone voltage, and collision energy

Kind	Excimer ion	Characteristic ion	Retention time (s)	cone voltage (V)	collision energy (eV)
Cobalamin	678.6	147.1	0.162	32	36
	678.6	443.4	0.162	32	20
Riboflavin	377.3	243.0	0.022	40	22
	377.3	172.1	0.022	40	37

Abbreviations used in Supplementary Information I

beta Net Relatedness Index: βNRI, beta mean nearest taxon distance: βMNTD, beta nearest taxon index: β NTI, homogeneous selection: HoS, heterogeneous selection: HeS, homogenizing dispersal: HD, dispersal limitation: DL, drift: DR, decision curve analysis: DCA, canonical correlation analysis: CCA, redundancy analysis: RDA, link test for environmental filtering: LTEF, moisture content: MC, electrical conductivity: EC, germination index: GI, mean squared error: MSE, within-module connectivity: Z_i value, among-module connectivity: P_i value, ribosomal RNA operons: rrn, Ribosomal RNA Operon Copy Number Database: rrndb, mean copy number: MCN, multiple linear regression: MLG, bayesian information criterion: BIC, Sequence Read Archive: SRA, Total carbon: TC.

Supplementary References

1. Anderson, M. J. A new method for non-parametric multivariate analysis of variance. *Austral Ecol*. **26**, 32-46 (2001).

2. Oksanen, J., Blanchet, F. G., Kindt, R., Legendre, P. & Wagner, H. Vegan: Community Ecology Package. R package version 2.0-3. R Foundation for Statistical Computing, Vienna, Austria., (2012).

3. Ning, D., Yuan, M., Wu, L., Zhang, Y., Guo, X. & Zhou, X. et al. A quantitative framework reveals ecological drivers of grassland microbial community assembly in response to warming. *Nat Commun*. **11**, (2020).

4. Stegen, J. C., Lin, X., Fredrickson, J. K., Chen, X., Kennedy, D. W. & Murray, C. J. et al. Quantifying community assembly processes and identifying features that impose them. *Isme J*. **7**, 2069-2079 (2013).

5. Zhou, J. & Ning, D. Stochastic community assembly: Does it matter in microbial ecology? *Microbiol Mol Biol R*. **81**, (2017).

6. Herren, C. M. & McMahon, K. D. Cohesion: A method for quantifying the connectivity of microbial communities. *Isme J*. **11**, 2426-2438 (2017).

7. D'Amen, M., Mod, H. K., Gotelli, N. J. & Guisan, A. Disentangling biotic interactions, environmental filters, and dispersal limitation as drivers of species co-occurrence. *Ecography*. **41**, 1233-1244 (2018).

8. Barner, A. K., Coblentz, K. E., Hacker, S. D. & Menge, B. A. Fundamental contradictions among observational and experimental estimates of non-trophic species interactions. *Ecology*. **99**, 557-566 (2018).

9. Yuan, M. M., Guo, X., Wu, L., Zhang, Y., Xiao, N. & Ning, D. et al. Climate warming enhances microbial network complexity and stability. *Nat Clim Change*. **11**, 100-343 (2021).

10. Trivedi, P., Delgado-Baquerizo, M., Trivedi, C., Hu, H., Anderson, I. C. & Jeffries, T. C. et al. Microbial regulation of the soil carbon cycle: Evidence from gene-enzyme relationships. *Isme J.* **10**, 2593-2604 (2016).

11. Jiao, S., Chen, W., Wang, J., Du, N., Li, Q. & Wei, G. Soil microbiomes with distinct assemblies through vertical soil profiles drive the cycling of multiple nutrients in reforested ecosystems. *Microbiome*. **6**, (2018).

12. Sunagawa, S., Coelho, L. P., Chaffron, S., Kultima, J. R., Labadie, K. & Salazar, G. et al. Structure and function of the global ocean microbiome. *Science*. **348**, (2015).

13. Deng, Y., Jiang, Y., Yang, Y., He, Z., Luo, F. & Zhou, J. Molecular ecological network analyses. *Bmc Bioinformatics*. **13**, (2012).

14. Olesen, J. M., Bascompte, J., Dupont, Y. L. & Jordano, P. The modularity of pollination networks. *P Natl Acad Sci Usa*. **104**, 19891-19896 (2007).

15. Weiss, S., Van Treuren, W., Lozupone, C., Faust, K., Friedman, J. & Deng, Y. et al. Correlation detection strategies in microbial data sets vary widely in sensitivity and precision. *Isme J.* **10**, 1669-1681 (2016).

16. Roller, B. R. K., Stoddard, S. F. & Schmidt, T. M. Exploiting rRNA operon copy number to investigate bacterial reproductive strategies. *Nat Microbiol*. **1**, (2016).

17. Abreu, C. I., Dal Bello, M., Bunse, C., Pinhassi, J. & Gore, J. Warmer temperatures favor slower-growing bacteria in natural marine communities. *Sci Adv*. **9**, e8352 (2023).

18. Croft, M. T., Warren, M. J. & Smith, A. G. Algae need their vitamins. *EUKARYOTIC CELL*. **5**, 1175-1183 (2006).

19. Banerjee, R. V. & Matthews, R. G. Cobalamin-dependent methionine synthase. *Faseb J.* **4**, 1450-1459 (1990).

20. De Gregoris, T. B., Aldred, N., Clare, A. S. & Burgess, J. G. Improvement of phylum- and class-specific primers for real-time PCR quantification of bacterial taxa. *J Microbiol Meth*. **86**, 351-356 (2011).
