# Supplementary material for: Inter-bacterial mutualism promoted by public goods in a system characterized by deterministic temperature variation
Source: Nat Commun. 2023 Sep 5;14:5394. doi: 10.1038/s41467-023-41224-7 (PMC10480208; doi:10.1038/s41467-023-41224-7)
Supplement: Supplementary file 3 — Description of Additional Supplementary Files [file 41467_2023_41224_MOESM3_ESM.pdf]

Description of Additional Supplementary Files:

Supplementary Data 1: Functional genes copy number

Supplementary Data 2: Key ASVs screening

Supplementary Data 3: Topological properties for constructed network based on 10 sample imputs that represent the average of all 6 time points for a given pile.

Supplementary Data 4: Relative abundance of neighboring ASVs (%)

Supplementary Data 5: Obtained MAGs

Supplementary Data 6: High quality MAGs

Supplementary Data 7: Relative abundance and activity of 159 MAGs

Supplementary Data 8: Cobalamin requirements of microbes related to the nitrogen cycle

Supplementary Data 9: Cobalamin requirements of microbes related to the cellulose degradation

Supplementary Data 10: Identification of microbial interaction type

Supplementary Data 11: Environmental factors

Supplementary Data 12: Primers for high-throughput qPCR

Supplementary Data 13: Topological parameters of 6 constructed network based on 60 sampling timepoints
