# Supplementary material for: Inter-bacterial mutualism promoted by public goods in a system characterized by deterministic temperature variation
Source: Nat Commun. 2023 Sep 5;14:5394. doi: 10.1038/s41467-023-41224-7 (PMC10480208; doi:10.1038/s41467-023-41224-7)
Supplement: Supplementary file 5 — Reporting Summary [file 41467_2023_41224_MOESM5_ESM.pdf]

nature portfolio

Corresponding author(s): Baolan Hu

Last updated by author(s): Aug 18, 2023

Reporting Summary

Nature Portfolio wishes to improve the reproducibility of the work that we publish. This form provides structure for consistency and transparency in reporting. For further information on Nature Portfolio policies, see our Editorial Policies and the Editorial Policy Checklist.

Statistics

For all statistical analyses, confirm that the following items are present in the figure legend, table legend, main text, or Methods section.

n/a	Confirmed	
☐	☒	The exact sample size (*n*) for each experimental group/condition, given as a discrete number and unit of measurement
☐	☒	A statement on whether measurements were taken from distinct samples or whether the same sample was measured repeatedly
☐	☒	The statistical test(s) used AND whether they are one- or two-sided<br>*Only common tests should be described solely by name; describe more complex techniques in the Methods section.*
☐	☒	A description of all covariates tested
☐	☒	A description of any assumptions or corrections, such as tests of normality and adjustment for multiple comparisons
☐	☒	A full description of the statistical parameters including central tendency (e.g. means) or other basic estimates (e.g. regression coefficient) AND variation (e.g. standard deviation) or associated estimates of uncertainty (e.g. confidence intervals)
☐	☒	For null hypothesis testing, the test statistic (e.g. *F*, *t*, *r*) with confidence intervals, effect sizes, degrees of freedom and *P* value noted<br>*Give P values as exact values whenever suitable.*
☒	☐	For Bayesian analysis, information on the choice of priors and Markov chain Monte Carlo settings
☒	☐	For hierarchical and complex designs, identification of the appropriate level for tests and full reporting of outcomes
☐	☒	Estimates of effect sizes (e.g. Cohen's *d*, Pearson's *r*), indicating how they were calculated

Our web collection on statistics for biologists contains articles on many of the points above.

Software and code

Policy information about availability of computer code

Data collection	No software was used for data collection.
Data analysis	Divisive Amplicon Denoising Algorithm 2 (DADA2) and QIIME2 (v2020.11.0) were used to process the high-throughput sequencing data. Trimmomatic (v.0.36), MEGAHIT (v1.0.6), MetaBAT2, CheckM, Genome Taxonomy Database Toolkit (GTDB-Tk; v 1.7.0), Bowtie2 (v2.33), and Corset (v1.06) were used to process the metagenomic and metatranscriptomics sequencing data. The R script for screening for screening overall abundant ASVs, ubiquitous ASVs, frequently abundant ASVs and PosCoh related ASVs is publicly available on GitHub at https://github.com/Yuxiang-Zhao/Composting or under Zenodo at https://doi.org/10.5281/zenodo.8260129.

For manuscripts utilizing custom algorithms or software that are central to the research but not yet described in published literature, software must be made available to editors and reviewers. We strongly encourage code deposition in a community repository (e.g. GitHub). See the Nature Portfolio guidelines for submitting code & software for further information.

Data

Policy information about availability of data

All manuscripts must include a data availability statement. This statement should provide the following information, where applicable:

- Accession codes, unique identifiers, or web links for publicly available datasets
- A description of any restrictions on data availability
- For clinical datasets or third party data, please ensure that the statement adheres to our policy

The 16S rRNA gene high-throughput sequencing, metagenomic sequencing, and metatranscriptomics sequencing data generated in this study have been deposited

in the National Center for Biotechnology Information (NCBI) Sequence Read Archive (SRA) database under under accession number PRJNA877822 [https://www.ncbi.nlm.nih.gov/bioproject/PRJNA877822], PRJNA878660 [https://www.ncbi.nlm.nih.gov/bioproject/PRJNA878660], and PRJNA897831 [https://www.ncbi.nlm.nih.gov/bioproject/PRJNA897831]. The database used for rrn copy number estimation is available online (https://rrndb.umms.med.umich.edu/). Source data are provided as a Source Data file.

Research involving human participants, their data, or biological material

Policy information about studies with human participants or human data. See also policy information about sex, gender (identity/presentation), and sexual orientation and race, ethnicity and racism.

Reporting on sex and gender	N/A
Reporting on race, ethnicity, or other socially relevant groupings	N/A
Population characteristics	N/A
Recruitment	N/A
Ethics oversight	N/A

Note that full information on the approval of the study protocol must also be provided in the manuscript.

Field-specific reporting

Please select the one below that is the best fit for your research. If you are not sure, read the appropriate sections before making your selection.

☐ Life sciences ☐ Behavioural & social sciences ☒ Ecological, evolutionary & environmental sciences

For a reference copy of the document with all sections, see nature.com/documents/nr-reporting-summary-flat.pdf

Ecological, evolutionary & environmental sciences study design

All studies must disclose on these points even when the disclosure is negative.

Study description	A quasi-natural community (composting) in a biosystem was monitored, where temperature (20℃-70℃) was the main abiotic stress. 10 piles were tested as independent replicates. Sampling for each pile was carried out on days 0, 5, 12, 20, 25, 30 depending on the changes of temperature. 16S rRNA gene high-throughput sequencing, metagenomic sequencing, and metatranscriptomics sequencing were combined to reveal the potential microbial cooperation in composting and identify the ACT ASVs. We further tested 3000 bacterial pairings to confirm the results obtained by sequencing methods. We found that mutualism was common (~39.1%) and competition was rare (~13.9%) in the pairs involving Thermobifida fusca and Saccharomonospora viridis. Our work offers a deep insight into how high temperature selected bacteria to favor mutualism and mask competition at both the community and species levels.
Research sample	The research samples were sampled from a food waste (FW) composting facility located in Zhejiang, China (30°52′51.50″N; 119°46′12.87″E). We monitored 10 piles (between May and August 2020) to reveal the bacterial community in food waste composting. Sampling was carried out on days 0, 5, 12, 20, 25, 30 depending on the changes of temperature, which is the deterministic factor in this quasi-natural system. These samples could represent the succession of bacterial community in a system characterized by deterministic temperature variation.<br>The 16S rRNA gene high-throughput sequencing, metagenomic sequencing, and metatranscriptomics sequencing data generated in this study have been deposited in the National Center for Biotechnology Information (NCBI) Sequence Read Archive (SRA) database under under accession number PRJNA877822 [https://www.ncbi.nlm.nih.gov/bioproject/PRJNA877822], PRJNA878660 [https://www.ncbi.nlm.nih.gov/bioproject/PRJNA878660], and PRJNA897831 [https://www.ncbi.nlm.nih.gov/bioproject/PRJNA897831].
Sampling strategy	To eliminate heterogeneity, each sample consisted of five sub-samples, including the four corners and the center of the pile. The samples were collected based on the changes of temperature, which is the deterministic driving force in composting. A total of 60 samples were included in the analyses, which is sufficient to represent the bacterial community diversities in food waste composting in this area and to allow statistical evaluation of the hypothesis we tested.<br>As Firmicutes is the dominant functional phylua in composting, we isolated 40 species affiliated with Firmicutes. We tested how Thermobifida fusca and Saccharomonospora viridis influenced them and used 3 high abundance species as control. We tested 3000 bacterial pairings, which is sufficient to represent the interaction between T. fusca and S. viridis and Firmicutes and to allow statistical evaluation of the hypothesis we tested.
Data collection	Total DNAs and RNAs were extracted using a standardized approach by Yuxiang Zhao. The V4 region of the bacterial 16S rRNA gene, metagenomic, and metatranscriptomics were sequenced by MAGIGENE Biological Technology Co. Ltd Guangzhou. Temperature was recorded everyday. Other physicochemical properties were measured by Yuxiang Zhao and Jingjie Cai. Experiments for bacterial pairing and measurement of cobalamin were conducted by Yuxiang Zhao, Jingjie Cai and Baofeng Zhang
Timing and spatial scale	Samples were taken from 10 composting piles (between May and August 2020). The sampling time points were set as follows: Day 00 (initial of composting), Day 05 (maximum mean temperature), Day 12 (halfway point between Day05 and Day20), Day 20 (last day with a mean temperature over 50℃), Day 25 (halfway point between Day20 and Day30), Day 30 (last day of composting). These

	samples covered the heating to cooling process in composting. The global presence of the ACT ASVs were detected by searching the Sequence Read Archive (SRA) with the 16S rRNA gene sequences by Integrated Microbial Next Generation Sequencing (IMNGS). 3000 bacterial pairings were conducted from February to August 2022.
Data exclusions	No data were excluded from the analyses.
Reproducibility	We tested 10 piles were as independent replicates. All relevant experiments were also performed in three technical replications (e.g., environmental factors, 16S rRNA gene high-throughput sequencing, metagenomic sequencing, and metatranscriptomics sequencing). An unexpected secondary warming occurred in Pile C. As Pile C completed the composting process, we did not exclude these data in order to respect the unexpected phenomena and exceptional samples.<br>We also confirm our results in in vitro work. To confirm the reproducibility, the tested strains were purchased from Deutsche Sammlung von Mikroorganismen und Zellkulturen (DSMZ, German) and checked by comparative genomics. All the replication showed a similiar results.
Randomization	Samples were divided into 6 groups by sampling times for following analyses (i.e., Day00 group, Day05 group, Day12 group, Day20 group, Day25 group, Day30 group).
Blinding	Data analysis was not blinded as treatment information were necessary to apply specific analysis.

Did the study involve field work? ☐ Yes ☒ No

Reporting for specific materials, systems and methods

We require information from authors about some types of materials, experimental systems and methods used in many studies. Here, indicate whether each material, system or method listed is relevant to your study. If you are not sure if a list item applies to your research, read the appropriate section before selecting a response.

Materials & experimental systems

n/a	Involved in the study
☒	☐ Antibodies
☒	☐ Eukaryotic cell lines
☒	☐ Palaeontology and archaeology
☒	☐ Animals and other organisms
☒	☐ Clinical data
☒	☐ Dual use research of concern
☒	☐ Plants

Methods

n/a	Involved in the study
☒	☐ ChIP-seq
☒	☐ Flow cytometry
☒	☐ MRI-based neuroimaging
